# New perspectives on head and neck allometry and ecomorphology in tetrapods

**DOI:** 10.1111/brv.70099

**Published:** 2025-11-11

**Authors:** Alice E. Maher, Philip G. Cox, Thomas W. Maddox, James D. Gardiner, Karl T. Bates

**Affiliations:** ^1^ Department of Musculoskeletal & Ageing Science Institute of Life Course & Medical Sciences, University of Liverpool William Henry Duncan Building, 6 West Derby Street Liverpool L7 8TX UK; ^2^ Centre for Integrative Anatomy, Department of Cell & Developmental Biology University College London Gower Street London WC1E 6BT UK; ^3^ School of Veterinary Science, Institute of Infection, Veterinary and Ecological Sciences, University of Liverpool, Small Animal Teaching Hospital Leahurst Campus, Chester High Road Neston CH64 7TE UK; ^4^ Department of Sport and Exercise Sciences, Faculty of Science and Engineering Manchester Metropolitan University Manchester M15 6BH UK

**Keywords:** skull, neck, allometry, evolution, trophic ecology, body size

## Abstract

The skull and neck are vital parts of the body, influencing feeding ecology, habitat exploitation and locomotion. Numerous studies have therefore sought to understand how the size of these segments vary with ecology and scale with overall body size. However, across past literature many different metrics have been used to represent both head and neck size, alongside disparate methods for body size normalisation and varied statistical approaches to analysing patterns. Furthermore, while several studies have examined allometric patterns across species of birds and dinosaurs, there are relatively few studies of other groups like mammals, non‐avian reptiles and amphibians. It is therefore currently difficult to combine or compare analyses from past studies to arrive at a clear picture of ecological and taxonomic trends in tetrapod head and neck allometry and evolution. To address these issues, we present a new analysis of head and neck proportions using a data set of 410 three‐dimensional digital skeletons that samples a wide taxonomic breadth of extinct and extant terrestrial tetrapods. Allometric and ecological patterns in head and neck size were analysed using phylogenetically informed approaches, with head and neck size quantified using multiple metrics representative of a range of methods used across previous studies. We find that different measurements used in the literature to represent head and neck size do not always yield qualitatively consistent results in terms of allometric patterns within and between major taxonomic and ecological groups. For example, across tetrapods, all metrics suggest negative allometry in skull size, whereas the pattern of allometry seen in the neck is influenced by the metric used (length *versus* volume). We also find that allometric patterns in linear metrics for head and neck size are better described by a linear model, whilst volumetric measurements are better fitted by a quadratic model for both the head and neck. Statistical support for quadratic models appears to be driven by species over 100 kg tending to show greater negative allometry in skull volume, whilst the neck shows strong positive allometry above this approximate size threshold. The disparate allometric patterns given by different metrics typically result from systematic variation in segment shape, which may often have adaptive significance. For example, distinct allometric trends in skull length and width are recovered across taxonomic and trophic groups, which may represent mechanical interactions between bite force and velocity in different feeding modes, particularly carnivory, insectivory and piscivory. Potentially adaptively significant patterns were also recovered in neck allometry for piscivores, and in the neck–head scaling seen in carnivores and herbivores, where a larger head in bigger carnivores enables capture of large prey but necessitates a reduced neck, while relatively smaller head sizes in herbivores (as food processing shifts to the gut) allow longer necks to increase the three‐dimensional volume (‘feeding envelope’) accessible to the head–neck system. The disparate qualitative and quantitative allometric relationships given by different metrics suggests that future work should carefully consider the choice of parameter used to represent skull and neck size when comparing trophic and taxonomic groups and making ecological and macroevolutionary inferences.

## I. INTRODUCTION

The structure and function of the head and neck in tetrapods has been the subject of considerable academic interest for a long time (Darwin, 1872; Westoll, 1943; Emerson, 1985; Christiansen, 1999; Xu *et al*., 2002; Goswami, 2006; Snively & Russell, 2007; Cox, 2008; Kulemeyer *et al*., 2009; Slater & Van Valkenburgh, 2009; Sander *et al*., 2011; Baab *et al*., 2014; Böhmer, Rauhut & Wörheide, 2015; VanBuren, Campione & Evans, 2015; Arnold, Amson & Fischer, 2017; Felice *et al*., 2019; Terray *et al*., 2020; Marek *et al*., 2021). The head is an important part of the body, containing the brain and sensory organs that together act as a processing centre for the animal. The head therefore has an important relationship with brain size (Henneberg, 1998), sexual selection (Gould, 1974; Weston, Friday & Liò, 2007; Rackovic *et al*., 2019; Morris *et al*., 2020), habitat use and locomotion (Trueb & Alberch, 1985; Herrel *et al*., 2008; Vanhooydonck *et al*., 2011; Fabre *et al*., 2016), and feeding ecology (Emerson, 1985; Christiansen, 1999; Goswami, 2006; Cox, 2008; Kulemeyer *et al*., 2009; Slater & Van Valkenburg, 2009; Baab *et al*., 2014; Felice *et al*., 2019). Feeding ecology, in particular, has been shown to influence the gross size and structure of the skull through deep time (Goswami, 2006; Cox, 2008; Kulemeyer *et al*., 2009; Slater & Van Valkenburg, 2009; Baab *et al*., 2014; Dumont *et al*., 2016); for example, a relatively larger skull allows for larger muscles and thus higher bite forces, which facilitate increased foraging efficiency and net energy intake (Herrel, De Grauw & Lemos‐Espinal, 2001; Verwaijen, Van Damme & Herrel, 2002; Van der Meij & Bout, 2006; Anderson, McBrayer & Larson, 2008; Law *et al*., 2018), affording animals the ability to consume otherwise unobtainable resources (Herrel, McBrayer & Larson, 2007; Anderson *et al*., 2008; Bulté, Gravel & Blouin‐Demers, 2008; Santana, Dumont & Davis, 2010; Gignac & Erickson, 2016).

The head of terrestrial vertebrates must be fully supported by the neck, which in all tetrapods is an intrinsic link between the head and the body and morphologically defined as the association of multiple cervical vertebrae (Romer, 1950). The neck is a complex structure and must be able to provide adequate support and rotation of the head about the spine to aid in a variety of actions such as feeding (Sander *et al*., 2011; Arnold *et al*., 2017; Böhmer *et al*., 2019; Marek *et al*., 2021), manipulation of objects (Dilger, 1960), combat (Senter, 2007; Laurin, 2010; Vander Linden & Dumont, 2019; Guevara & Hurme, 2019), sexual display (Simmons & Scheepers, 1996; Senter, 2007; Bohmer *et al*., 2019) and stabilisation of the head during locomotion (Warrick, Bundel & Dial, 2002; Pete *et al*., 2015; Marek *et al*., 2021). The fundamental role of the neck in maintaining functionality of the head means that the evolutionary ecomorphologies of these two body regions have often been said to be intrinsically linked. For example, changes in neck length have been observed to correlate with ecologically adaptive changes in head size (e.g. Bohmer *et al*., 2019; Marek *et al*., 2021; Arnold, 2021; Maher *et al*., 2022).

Evolutionary variation in neck length and size can be achieved by adding or removing cervical vertebrae. For example, amphibians have only one cervical vertebra (Vidal, Graf & Berthoz, 1986; Wake, 2009) while long‐necked sauropod dinosaurs often had in excess of 15 (Taylor & Wedel, 2013). Overall neck length can also vary due to variation in the size of individual vertebrae. With a fixed set of seven cervical vertebrae, neck elongation in mammals (exemplified by species with exceptionally long necks such as giraffes) is achieved through the elongation of each individual cervical vertebra (Galis, 1999; Narita & Kuratani, 2005; Arnold, 2021) and recently a similar mode of variation was proposed to underpin the highly disparate neck lengths seen in birds (Marek *et al*., 2021).

Across tetrapods, head and neck size vary considerably. Identifying the factors that drive evolutionary changes in the heads and necks of tetrapods has been a challenge because of the difficulties in quantifying complex morphologies, sampling a broad number of taxa and identifying mechanisms responsible for generating macroevolutionary patterns. In this study, the methods for quantifying and analysing head and neck size evolution in terrestrial tetrapods are surveyed, alongside current views on allometric patterns present in major taxonomic and trophic groups. This review highlights that previous studies have used a variety of metrics to quantify head and neck size in relation to body size in different tetrapod subgroups and have subsequently employed different size‐normalisation and statistical techniques to examine macroevolutionary patterns. This currently makes it challenging to compare results across studies, and in some cases to compare adaptive trends seen in different vertebrate clades.

To begin to address these issues, a new analysis of head and neck proportions is presented using a data set of 410 three‐dimensional (3D) digital skeletons that widely samples a considerable taxonomic breadth of extinct and extant terrestrial tetrapods. Head and neck size in this data set are quantified using multiple metrics representative of a range of methods employed across previous studies and subsequently used to analyse allometric and ecological patterns using phylogenetically informed statistical approaches. Analysis of this data set allows us to examine how different tetrapod groups scale by different segment size metrics, providing new information on dietary and taxonomic subgroups, in addition to methodological insights on how the choice of metric used to represent the head and neck impacts biological and macroevolutionary interpretations.

## II. QUANTIFYING HEAD AND NECK SIZE: A REVIEW OF MEASUREMENTS AND METHODS

### (1) Linear measurements

#### (a) Head size

**Fig. 1:**
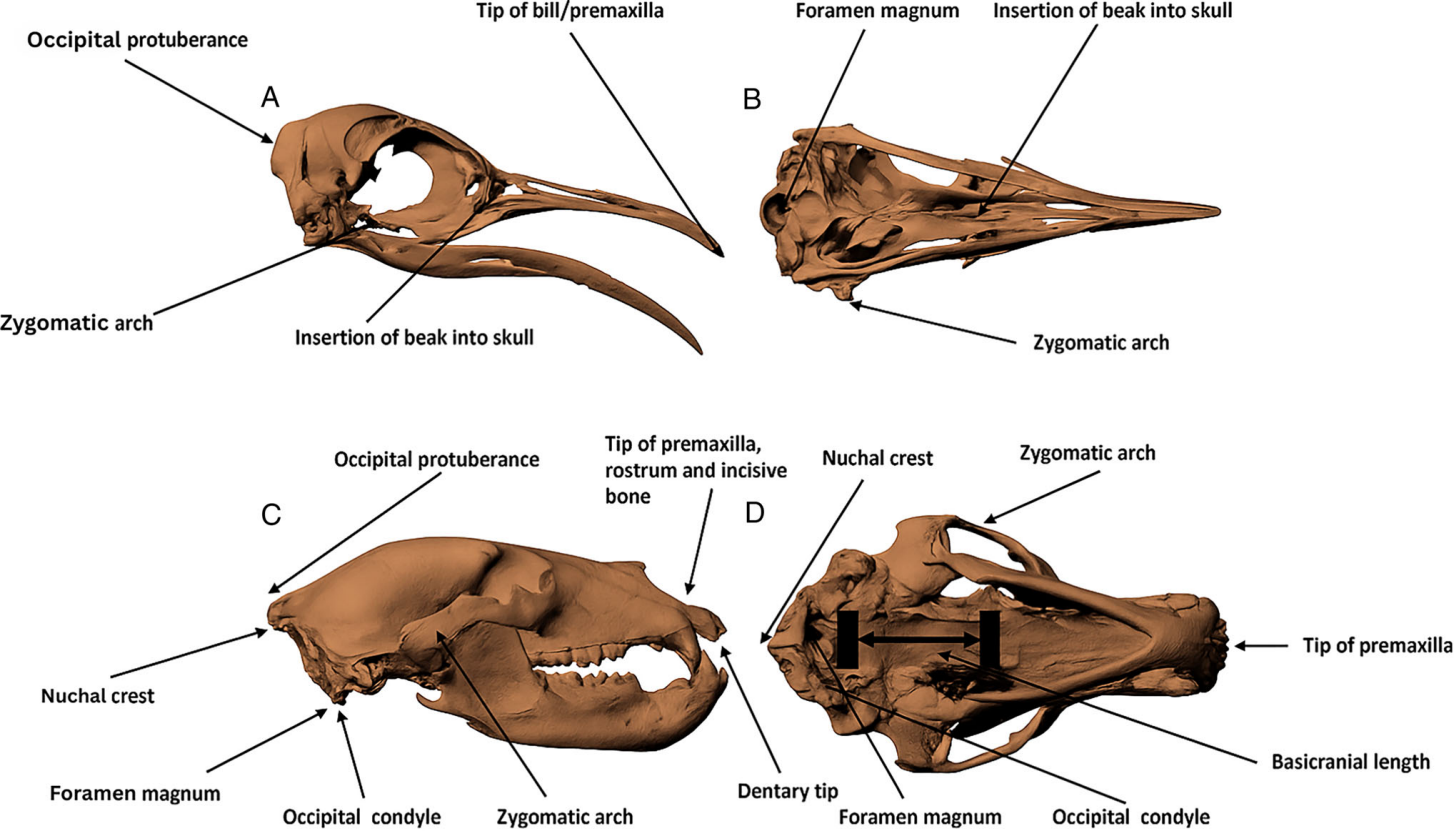
Measuring tetrapod skull size. Common anatomical areas on the skulls of (A, B) birds and (C, D) mammals used to measure skull length and width in the literature. Left panels (A and C) represent lateral views, while right panels (B and D) are ventral views. Species represented are *Aptenodytes forsteri* and *Ursos arctos*.

Previous studies have used various measures such as length, width, or height as a proxy for head mass and/or the overall size of the head (e.g. Christiansen, 1999; Van der Leeuw, 2002; Sereno *et al*., 2007; McGarrity, Campione & Evans, 2013). Head length in many animal groups is generally defined as the greatest length of the skull from the most posterior to the most anterior point, usually the distance between the occipital condyle and the anterior‐most projection (e.g. Gould, 1974; Radinsky, 1981; Emerson, 1985; Birch, 1999; Erickson, Lappin & Vliet, 2003; Christiansen & Adolfssen, 2005; Therrien & Henderson, 2007; Jones, 2008; Lappin *et al*., 2017). However, in some animal groups there is variation in the precise approach used to measure skull length (Fig. 1). For example, in mammals, Lieberman, Pearson & Mobray (2000) used basicranial length suggested by Radinsky (1984) as a proxy for skull length to avoid the confounding effects of jaw length variation that can arise when total skull length is used. By contrast, Choudhary *et al*. (2020) used the distance between the highest points of the parietal bones to the rostral margin of the premaxilla, whilst Timm‐Davis, DeWitt & Marshall (2015) measured the maximum length of the skull from the tip of the rostrum to the nuchal crest (Fig. 1). Similar differences in approach can also be seen in birds, where skull length has been measured as the greatest length from the tip of the bill to the most caudal end of the skull (e.g. Hallgrimsson *et al*., 2016; Kass, Montalti & Hospitaleche, 2018; Süzer *et al*., 2018; Angst *et al*., 2020), from the occipital protuberance to the distal tip of the premaxilla (e.g. Thompson *et al*., 1999; Verdiglione & Rizzi, 2018), the foramen magnum to the tip of the bill (Setiawan, Darby & Lambert, 2004), and from the occipital bone to the insertion of the beak into the skull (Francesch, Villalba & Cartañà, 2011) (Fig. 1).

In contrast to the subtle methodological variations seen in measurements of skull length, head width is nearly universally measured as the greatest width of the skull, which is generally the maximum distance between the two zygomatic arches, across all species including mammals (Gould, 1974; Radinsky, 1981; Timm‐Davis *et al*., 2015; Choudhary *et al*., 2020), birds (Süzer *et al*., 2018; Verdiglione & Rizzi, 2018), reptiles (Herrel & O'Reilly, 2006) and dinosaurs (Christiansen, 1999). In extant amphibians where the zygomatic arch is not present, skull width has also been measured as the widest part of the skull, which is the lateral extent of the jaw joints (Emerson, 1985; Lappin *et al*., 2017).

#### (b) Neck size

Most studies calculate neck size as the sum of centrum lengths from individual cervical vertebrae regardless of the taxonomic group under study (e.g. Marek *et al*., 2021; Böhmer *et al*., 2019; Arnold *et al*., 2017; Dzemski & Christian, 2007; O'Keefe & Hiller, 2006; Taylor & Wedel, 2013). Many measurements in the literature for neck length do not consider the contribution of cartilage and intervertebral discs to neck length as measurements are generally taken from skeletons and/or fossils (Böhmer *et al*., 2019; Arnold *et al*., 2017; O'Keefe & Hiller, 2006; Taylor & Wedel, 2013). However, for some extant species neck lengths have been measured by adding in the length of cartilage and intervertebral disc lengths (Dzemski & Christian, 2007). The contribution of unpreserved joint tissues is difficult to account for in extinct species and where intervertebral disc length has been estimated in sauropod species it results in relatively large changes to the estimated length of the neck (Taylor & Wedel, 2013).

The cross‐sectional properties of the neck not only contribute to gross neck size, but also play an important but largely understudied role in neck mechanical function. In their conceptual discussion of sauropod dinosaur neck construction and function, Preuschoft & Klein (2013) note the importance of neck cross‐sectional geometry in determining the torsional capabilities of vertebrate necks and note that, at least qualitatively, the cylindrical cross‐sectional shape of sauropod necks may have increased inherent torsional strength and facilitated three‐dimensional rotation. To our knowledge, there have been no large‐scale quantitative assessments of geometric and allometric trends in cross‐sectional neck properties across major taxonomic and ecological tetrapod groups.

### (2) Mass and volumetric measurements

**Fig. 2:**
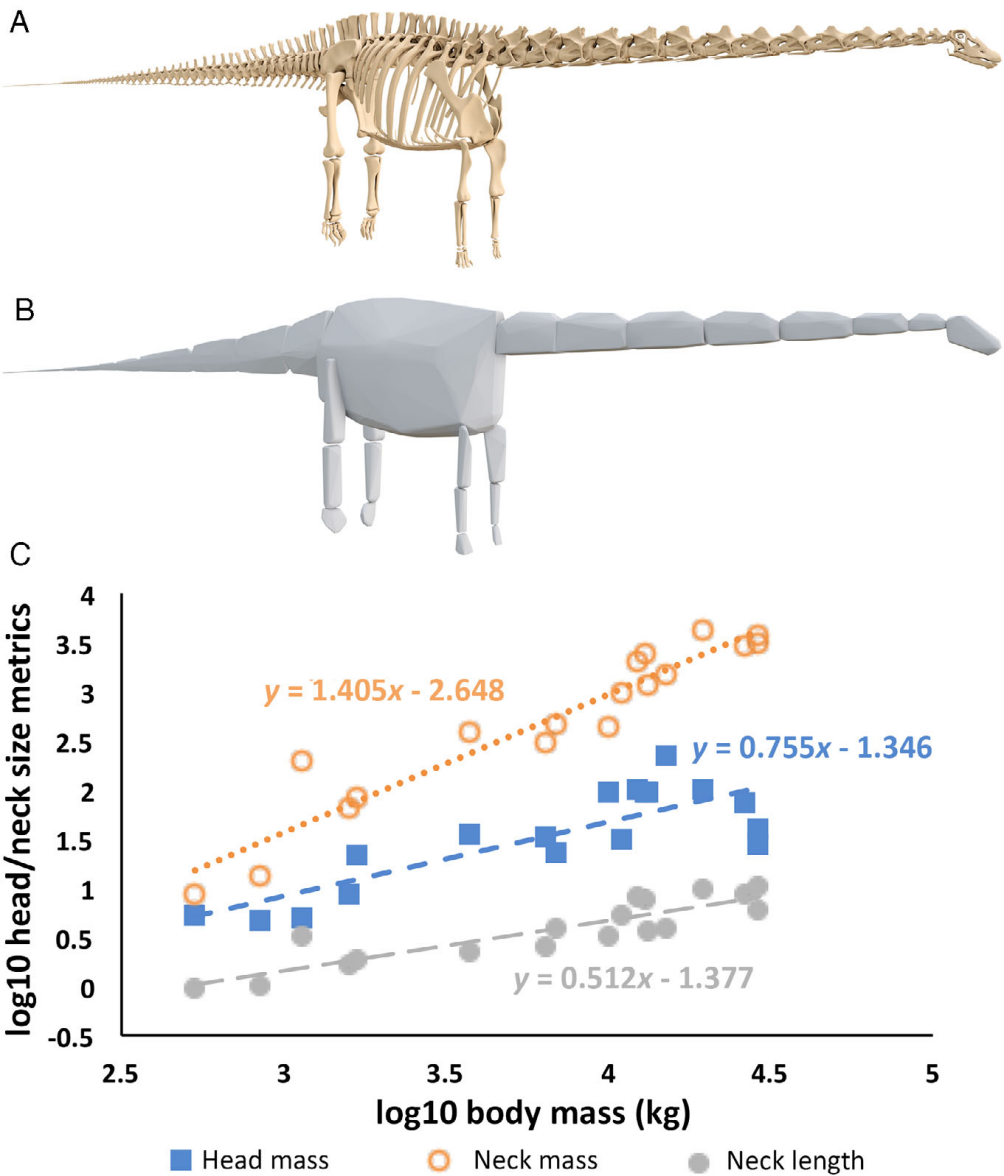
Volumetric modelling of sauropodomorph dinosaur body size and relative head and neck size. (A, B) Bates *et al*. (2016) used volumetric modelling (minimum convex hulling approach) to estimate total body mass and individual body segment masses in sauropodomorph dinosaurs. (C) Analysis of their data suggests their approach recovers positive allometry in neck mass (orange circles; for isometry, expected slope = 1) and neck length (grey circles; for isometry, expected slope = 0.33) but negative allometry in head mass (blue squares; for isometry, expected slope = 1) with respect to body mass.

An alternative approach to using linear measurements of the head and neck as a proxy for overall segment size is to use a volumetric approach (Fig. 2). This can be achieved through physical scale models (e.g., Gregory, 1905; Colbert, 1962; Alexander, 1985) or 3D virtual models (e.g. Allen, Paxton & Hutchinson, 2009; Allen *et al*., 2013; Sellers *et al*., 2012; Henderson, 2013; Brassey & Gardiner, 2015; Bates *et al*., 2009a,b, 2012, 2015, 2016, 2025; Snively *et al*., 2013; Brassey & Sellers, 2014; Maidment, Henderson & Barrett, 2014; Brassey, 2016; Macaulay, Hutchinson & Bates, 2017; Marek *et al*., 2021; Maher *et al*., 2022; Macaulay *et al*., 2023). Briefly, these approaches either directly extract skin volume from medical image data (e.g. Allen *et al*., 2009; Coatham, Sellers & Püschel, 2021; Cross *et al*., 2024; Macaulay *et al*., 2017, 2023) or estimate it through manual sculpture (e.g. Allen *et al*., 2013; Bates *et al*., 2009a,b; Bishop *et al*., 2020; Henderson, 2013; Hutchinson *et al*., 2011) or mathematical shape‐fitting (e.g. Sellers *et al*., 2012; Brassey & Gardiner, 2015; Brassey *et al*., 2018; Marek *et al*., 2021; Maher *et al*., 2022; Macaulay *et al*., 2023; van Biljert *et al*., 2024; Bates *et al*., 2015, 2016, 2025; Dempsey *et al*., 2025a,b). This volumetric measure is either then used itself analytically or converted to a mass through multiplication by a density value depending on the goal of the analysis. While hardware and software advances (and cost effectiveness) have made digitisation techniques more accessible in recent decades (e.g. Bates *et al*., 2010; Falkingham, 2012), volumetric approaches rely upon more complete skeletal material and typically require far more labour to carry out compared to linear measurements (Campione & Evans, 2020). As a result, volumetric approaches have tended to be used only in studies where a mass value was specifically required for the analysis undertaken (rather than a circumstance where any measure of gross head/neck size might suffice). Examples include assessment of the role of the head and neck in the evolution of whole‐body shape (e.g. Allen *et al*., 2013; Maidment *et al*., 2014; Bates *et al*., 2016; Maher *et al*., 2022; Macaulay *et al*., 2023; Dempsey *et al*., 2025b), estimation of aspects of organism thermal biology (e.g. Henderson, 2013), evaluation of the relationship between head mass and neck length allometries (e.g. Marek *et al*., 2021) and facilitation of biomechanical assessments of neck–head function (e.g. Snively *et al*., 2013).

### (3) Metrics for size normalisation

A measure of overall animal body size is required to assess allometric patterns and to normalise measures of head and neck size to evaluate relative similarities and differences in body proportions across animals. Arguably the most fundamental measure of the overall body size of an individual is their body mass and it is widely considered as the gold standard size‐normalisation metric (Campione & Evans, 2012, 2020), particularly in functional and biomechanical studies. As a result, many studies have used direct measures or estimates of body mass to normalise head and neck size for comparative purposes (e.g. Kleiber, 1947; Hemmingsen, 1960; Jerison, 1969; Peters, 1986; Brown, Marquet & Taper, 1993; Burness, Diamond & Flannery, 2001; Gillooly *et al*., 2001, 2002; Capellini & Gosling, 2007; McClain & Boyer, 2009). However, in many instances the body mass of specimens is unavailable to researchers. For example, the body mass of osteological specimens in museums is often not recorded, while for extinct species direct measures of mass are fundamentally impossible. In such instances, a variety of approaches have been utilised to derive representative measures of body mass to normalise head and neck size. By far the most common approaches are extant skeletal scaling relationships and volumetric reconstructions. A brief overview of the methodology, benefits, and limitations of these approaches is provided below. The reader is directed to Campione & Evans (2020), Brassey (2016) and Dempsey *et al*. (2025b) for more exhaustive reviews.

#### (a) Bone scaling approaches

At the simplest level, bone scaling approaches substitute a direct absolute measure for overall body size (e.g. body mass) with the absolute size of single bone or skeletal element. For example, Bestwick *et al*. (2022) used femur length as a proxy for overall body size in order to assess allometric patterns in skull size across Archosauria, while a study of turtle skull allometry used carapace length (Hermanson *et al*., 2022). In more complex analyses, predictive statistical relationships are constructed between the size of a bone or skeletal element and overall body mass, thereby allowing numerical estimation of body mass in other specimens where only that bone or skeletal element is available. Such regression‐based predictive models, based on the relationship between specific osteological measurements and body mass in extant animals, are the most common approach to estimating body mass in fossil specimens (e.g. Greenewalt, 1975; Campell Jr & Tonni, 1983; Damuth & MacFadden, 1990; Gingerich, 1990; Campbell & Marcus, 1992; Finarelli & Flynn, 2006; Butler & Goswami, 2008; Rinderknecht & Blanco, 2008; Millien & Bovy, 2010; Field *et al*., 2013; Ghizzoni, 2014). The popularity of this approach stems from its wide utility as it is possible to derive body mass from highly incomplete skeletal remains, compared to volumetric approaches, which require fairly complete skeletons (Campione & Evans, 2020). Most scaling approaches use elements involved in weight bearing during locomotion such as femoral and humeral circumference (Anderson, Hall‐Martin & Russell, 1985; Campione & Evans, 2012) or femoral head width (Ruff, Scott & Liu, 1991). Cranial metrics are also used (Wroe *et al*., 2003; Spoctor & Manger, 2007), but these can be highly variable especially for studies using taxa with diverse phylogenetic histories, which may lead to biases in final mass estimation if interspecific scaling patterns are not properly assessed (Damuth & MacFadden, 1990; Millien & Bovy, 2010).

Using regression‐based models therefore offers an efficient method with which to generate large data sets of body mass and subsequently for examining broad‐scale patterns of evolution (Laurin, 2004; Carrano, 2006; Finarelli & Flynn, 2006; Sookias, Butler & Benson, 2012; Benson *et al*., 2014, 2018; Puttick, Thomas & Benton, 2014; Kubo & Kubo, 2016). This approach is also relatively simple to use, as linear measurements can be taken using physical (calliper‐based) or digital morphometric methods requiring very little training, allowing for minimal user input and few subjective assumptions when applied to fossil specimens. As a statistical approach, this method also provides confidence or prediction intervals that can provide broad error bounds for use in macroevolutionary hypothesis testing, although actual application of this is relatively rare (Campione & Evans, 2012; Serrano, Palmqvist & Sanz, 2015).

#### (b) Volumetric methods

Volumetric methods (Fig. 2) approximate or estimate mass by multiplying a measured or estimated value for body volume by a value for average tissue density. The same diversity of physical and computational approaches to approximating or estimating body volume noted above for the head and neck have been applied to estimate overall body mass, particularly in extinct species. Historically, physical approaches were favoured (Colbert, 1962), but these have now largely been replaced by computational approaches to volume reconstruction. Within computational volumetric approaches there has been a gradual shift from manual volume sculpture, where an investigator subjectively decides upon the sizes and shapes of body volumes (e.g. Hutchinson, Ng‐Thow‐Hing & Anderson, 2007; Bates *et al*., 2009a,b, 2012; Henderson, 2013; Snively *et al*., 2013; Maidment *et al*., 2014), to more automated approaches where the final body volume is partially (e.g. Allen *et al*., 2009) or wholly guided by soft tissue proportions measured in living animals (e.g. Sellers *et al*., 2012; Brassey & Sellers, 2014; Bates *et al*., 2015, 2016, 2025; Coatham *et al*., 2021; van Biljert *et al*., 2024; Macaulay *et al*., 2023; Dempsey *et al*., 2025a,b). These approaches circumvent the single bone problem seen in regression analysis of isolated bone dimensions by including the maximum amount of information from the skeleton into the mass estimate. However, the approach is restricted by the need for relatively complete skeletons, which limits its capacity in larger scale macroevolutionary studies (Campione & Evans, 2020).

#### (c) Non‐linear allometry and differential scaling in small versus large animals

Whether ‘small’ animals scale differently than ‘large’ animals, and thus if there are size thresholds in morpho‐functional construction, is a fascinating question in organismal biology. Presently, studies that have quantitatively addressed the question of non‐linearity in organismal body segment allometry have tended to focus on a gross body size or shape metric, such as body length (e.g. Economos, 1983; Silva, 1998), or on the limb segments (e.g. Campione, 2017) given their importance for structural support and relevance to hypotheses surrounding the scaling of locomotor posture and performance (e.g. Biewener, 1989, 2005; Christiansen, 2002; Fuentes, 2016). To our knowledge, only Maher *et al*. (2022) have quantitatively assessed non‐linear allometry in the gross or overall size of the head and neck in tetrapods. They found slightly stronger statistical support for quadratic than linear scaling models of overall head and neck convex hull volume, suggesting that (at least when gross size in quantified in this way) these segments may scale non‐linearly in tetrapods as a whole. Interestingly, the pattern in this non‐linear allometry differs in the skull compared to the neck, with larger taxa tending towards relatively smaller skulls but longer necks, particularly above ~500 kg body mass (Maher *et al*., 2022). Whether similar patterns are recovered in more commonly used linear representations of head and neck size (lengths and/or widths) is currently unknown.

### (4) Overview of allometric and ecological studies of head and neck size

#### (a) Mammals

There appears to be a prevalent misconception in the literature regarding scaling patterns in the mammalian skull. For example, both Bestwick *et al*. (2022) and Arnold *et al*. (2017) cite a body of literature concerned with cranial shape as demonstrating isometric scaling of head size to overall body size in mammals as whole (Cardini & Polly, 2013; Cardini *et al*., 2015; Cardini, 2019), while Bestwick *et al*. (2022) also suggest similar studies show either isometry or positive allometry within specific mammalian subgroups (Slater & Van Valkenburgh, 2009; Tamagnini, Meloro & Cardini, 2017; Law *et al*., 2018). These cited studies (Cardini & Polly, 2013; Cardini *et al*., 2015; Cardini, 2019; Slater & Van Valkenburgh, 2009; Tamagnini *et al*., 2017; Law *et al*., 2018) provide key insights into cranial design in mammals, and how smaller skulls may differ systematically in shape to larger skulls, but they do not seek to assess patterns in skull size relative to body size, nor do they contain the data (e.g. measures of overall body size) required to do this. In Section V, we discuss the implications of these misconceptions on scaling patterns in mammal head size relative to other groups (Bestwick *et al*., 2022) and in the context of interpretations of selective pressures and mechanical constraints acting on head–neck allometry in tetrapods (Arnold *et al*., 2017).

To our knowledge, allometry of skull length relative to body size has only been quantitatively assessed in individual mammalian subgroups, but not across mammals as a whole (Table 1). All these studies recover negative allometry in skull length with respect to body mass. Carnivoran skulls were found to scale with negative allometry by Van Valkenburgh (1990), who used average body mass values for each species from the literature and ordinary least squares (OLS) regression without consideration of phylogenetic relationships. All families within this carnivoran data set also scaled with negative allometry (slopes 0.185–0.317, where isometric scaling equates to a slope of 0.33), although small sample sizes in some cases led to extremely broad confidence intervals that included isometry (e.g. Ursidae, slope 0.241 ± 0.101). A data set consisting only of Canidae and Ursidae also recovered negative allometry between skull length and literature‐derived average body masses (Figueirido *et al*., 2011). Janis (1990) did not present raw numerical values or the slope of their OLS relationship, but our manual digitisation of their graphs suggests unambiguous negative allometry in skull length in ungulates and macropodoids combined (slope: 0.302 ± 0.012). Again, standard OLS regression without consideration of phylogenetic relationships was used, with body mass represented by average species values from the literature (Janis, 1990). Rodent skulls have also been recovered as scaling with negative allometry relative to body mass in two independent studies (Rinderknecht & Blanco, 2008; Bertrand, Schillaci & Silcox, 2016).

**Table 1:** Summary of previous research on allometric scaling of head and neck size relative to body size and each other. Abbreviations: BM, measured specimen body mass; BL, body length; CL, carapace length; FL, femur length; HH, head height; HM, head mass; HW, head width; HV/M, head volume or mass; Lit. BM, literature‐derived body mass for each species; NL, neck length; NM, neck mass; SCS, skull centroid size (from geometric morphometric analysis); SE BM, scaling equation body mass; SL, skull length; SVL, snout–vent length; TibL, tibial length; TkL, trunk length; VolBM, body mass derived from a volumetric model.

Group/subgroup	Head ~ body size	Neck ~ body size	Head ~ neck size	Study
Mammals				
All	–	Negative (NL ~ Lit. BM; NL ~ TibL)	–	Arnold *et al*. (2017)
Rodents	Negative (SL ~ BM)	–	–	Bertrand *et al*. (2016)
Rodents	Negative (SL ~ BM)	–	–	Rinderknecht & Blanco (2008)
Canidae + Ursidae	Negative (SL ~ Lit. BM)	–	–	Figueirido *et al*. (2011)
Carnivorans	Negative (SL ~ Lit. BM)	–	–	Van Valkenburgh (1990)
Ungulates + macropodoids	Negative (SL ~ Lit. BM)	–	–	Janis (1990)
Birds				
All	Isometric (HV/M ~ SE BM)	Isometric (NL ~ SE BM)	Isometric (HM ~ NL)	Marek *et al*. (2021)
All	–	Isometric (NL ~ Lit. BM)	–	Böhmer *et al*. (2019)
Anseriformes	Negative (HL ~ BM)			van der Leeuw (2002)
Non‐avian dinosaurs				
All	Negative (SL ~ FL)	–	–	Bestwick *et al*. (2022)
Non‐avian theropods	Negative (SL ~ VolBM)	–	–	Therrien & Henderson (2007)
Non‐avian theropods	Positive (SL ~ BL)			Therrien & Henderson (2007)
Non‐avian theropods	Negative (SL ~ FL)	–	–	Bestwick *et al*. (2022)
Ceratopsians	Positive (SL ~ VolBM)	–	–	Sereno *et al*. (2007)
Ceratopsians	Negative (SL ~ FL)	–	–	Bestwick *et al*. (2022)
Sauropods	Negative (HM ~ VolBM)	Positive (NM ~ VolBM; NL ~ VolBM)	–	Bates *et al*. (2016)
Sauropods	Negative (SL ~ FL)	–	–	Bestwick *et al*. (2022)
Sauropods	–	Positive (NL ~ TkL)	–	Parrish, (2006)
Other reptiles				
Crocodyliformes	Isometric (HW ~ BM)			O'Brien *et al*. (2019)
Turtles	Negative (SCS ~ CL)			Hermanson *et al*. (2022)
Turtles	Isometric (HH ~ BM; HW ~ BM)			Marshall *et al*. (2014)
Amphibians				
All	–	–	–	–
Anurans (6 species)	Variable across species (HW ~ SVL)	–	–	Goldberg *et al*. (2024)

The skulls of carnivorous mammalian taxa tend to be short and wide with a large volume. These features enable the skull to increase the mechanical advantage of their jaw closing muscles and to deal with large torsional loadings from struggling prey (Radinsky 1981, 1984; Wroe & Milne, 2007; Felice, Pol & Goswami, 2021). Maher *et al*. (2022) also found an association between skull volume and carnivory in most taxonomic groups including mammals which exhibited the least strong negative allometry in skull volume, which may be related to hypercarnivory and large‐prey specialisation (Erickson *et al*., 2003; Anderson *et al*., 2008).

The mass of the vertebrate head must be supported by the neck and previous researchers have proposed that this may impose a mechanical constraint on relative head to neck length in tetrapods (Preuschoft & Klein, 2013; Arnold *et al*., 2017); for example the evolution of larger heads will tend to be causatively linked to reduced neck lengths in order to reduce the neck bending moments by bringing the larger head closer to the fulcrum of the neck. To our knowledge, only Arnold *et al*., (2017) quantified neck length allometry in mammals and concluded that mammalian necks do indeed scale with negative allometry relative to body mass when regression slopes considered phylogenetic relationships (Table 1), but with positive allometry when non‐phylogenetic regression was used. Because of the misconception that mammalian heads have been shown to scale isometrically, this finding was used to infer that that larger heads tend to evolve in concert with shorter necks, consistent with the theory that head mass actively enforces a mechanical constraint on neck length (Arnold *et al*., 2017).

#### (b) Birds

Previous studies have suggested either a negative allometric relationship between head length and body mass in anseriforms (Van der Leeuw, 2002) or an isometric relationship between head mass and body mass (Marek *et al*., 2021) in birds generally, but negative allometry when subgroups of birds were considered (Marek *et al*., 2021; Table 1). Independent studies have recovered similar allometric patterns in neck length. Marek *et al*. (2021) and Böhmer *et al*. (2019) recovered an isometric relationship between neck length and body mass (Table 1). The former study also recovered isometric scaling between the neck and head in birds, noting also an effect of insectivory on their allometric models, specifically that insectivorous birds have a generally longer neck than other birds (Marek *et al*., 2021). This isometric scaling of neck length with body mass and head mass differs from other groups of vertebrates: for example, negative allometry in neck length has been recovered in mammals (e.g. Arnold *et al*., 2017) where the weight of the head is hypothesised to serve as a constraint on neck length. It has been suggested that specific aspects of avian anatomy and biology, such as negative scaling of the brain and eye size with body mass, the reduction of jaw musculature and the widespread pneumatization of the skull, may have weakened the mechanical constraint on relative head–neck size that is commonly seen in mammals (Marek *et al*., 2021). Interestingly, there is good evidence that neck length scales isometrically with total hind‐limb length in birds, which may be the product of correlated evolution between these body regions related to the basic mechanical benefit of maintaining a neck length capable of allowing the head to reach and forage on the ground as hind‐limb length increases (Böhmer *et al*., 2019).

#### (c) Non‐avian dinosaurs

Competing analyses of non‐avian dinosaur scaling patterns have used different metrics to represent both head size and overall body mass and subsequently have yielded qualitatively different results (Table 1). For example, Therrien & Henderson (2007) suggested that skull length scales with negative allometry relative to body mass but positive allometry relative to body length in non‐avian theropod dinosaurs. Negative allometry was recovered by Bestwick *et al*. (2022) based on the relationship between skull length and femur length in non‐avian theropods. Sereno *et al*. (2007) found positive allometry in the skull length of ceratopsians, which contrasts with the negative allometry recovered in the data of Bestwick *et al*. (2022) and the isometric relationship that Bestwick *et al*. (2022) infer is present in the data of VanBuren *et al*. (2015). The contrasting results in the two former studies may be attributable to the use of difference metrics for overall body size, with Sereno *et al*. (2007) using body masses derived from volumetric models, while Bestwick *et al*. (2022) used femur length as their body size proxy. We are unable to see any formal assessment of allometry within ceratopsians in the study by VanBuren *et al*. (2015) and conclude that the assertion of Bestwick *et al*. (2022) that this study recovered isometric scaling of the skull represents a misinterpretation of their data. In sauropod dinosaurs, both Bates *et al*. (2016) (Fig. 2) and Bestwick *et al*. (2022) concluded that the head scaled with negative allometry, despite use of different metrics for both head and overall body size. To our knowledge, only one study has assessed the scaling of head to body size in Dinosauria as a whole and concluded that the group exhibited negative allometry (Bestwick *et al*., 2022).

Dinosaurs exhibit considerable variation in absolute and relative neck length (Gauthier, 1986; Sereno, 1991; Pisani *et al*., 2002). Like some groups of birds, sauropod dinosaurs had very long necks and possessed epiphyses similar to that of birds suggesting they were myologically similar (Taylor & Wedel, 2013). Neck length has been suggested to exhibit strong positive allometry in sauropods relative to torso length (Parrish, 2006) and estimated body mass (Bates *et al*., 2016; Fig. 2), where quadrupedality provided a platform for both large body sizes and longer necks, which likely increased the three‐dimensional volume (‘feeding envelope’) accessible to these herbivores (Taylor *et al*., 2011). Large non‐avian theropods, such as *Tyrannosaurus rex*, did not evolve necks as long as sauropods with similar mass, perhaps partly because the increased moment caused by neck elongation in a biped must be counteracted by an equal moment caused by a longer or more massive tail, increasing physiological cost. Another reason is due to the need for a larger head for killing prey. With a larger head a shorter neck provides stability for predation upon other large dinosaur species and a larger head allows more powerful musculature for wielding the jaws during feeding (Snively *et al*., 2013).

Previous work has suggested that a shift away from carnivory may have facilitated longer necks and smaller skull sizes in non‐avian dinosaurs (Zanno & Makovicky, 2011). A smaller head size in sauropods was possibly associated with a lack of dentition and the absence of oral food processing (Chure *et al*., 2010). This in turn may also have allowed for longer necks because of the reduction in head weight, reducing the power needed to lift the head and therefore reducing the required muscle mass (Taylor & Wedel, 2013; Taylor *et al*., 2011). However, within ornithischians such as ceratopsians, a massive dentition was needed for oral processing of their food which may have resulted in larger heads supported by shorter necks, or an intermediate head on an intermediate sized neck in hadrosaurs (Taylor & Wedel, 2013; Taylor *et al*., 2011). As a result of the large head size in ceratopsids, the centre of mass was located further anteriorly, implying that the evolution of a larger head size might also result in a structural need to revert to a quadrupedal pose (Maidment *et al*., 2014).

#### (d) Other reptiles

In non‐avian reptiles such as turtles and crocodylomorphs the skull has been inferred to scale either isometrically or with negative allometry (Table 1). For example, head width was found to scale isometrically with measured body mass in Crocodyliformes (O'Brien *et al*., 2019), while isometry in both head width and head height relative to body mass has been recovered for turtles and these trends have been correlated with allometric patterns in bite force (Marshall *et al*., 2014). However, Hermanson *et al*. (2022) suggested that skull size scaled with negative allometry, using carapace length rather than body mass as their measure of overall body size, and linking this finding to neck retraction capacity.

Less attention has been paid in other reptiles to the allometric relationship of the neck to body mass. However, within this taxonomic group feeding ecology almost certainly has a considerable impact on the size of the neck. For example, longer necks seen in fish‐eating reptiles and suction feeders such as in some freshwater turtle species is believed to allow for rapid acceleration of their heads for fast strikes towards their prey (Lemell *et al*., 2002; Hermanson *et al*., 2022).

#### (e) Amphibians

To our knowledge, allometric trajectories of the neck have not been formally assessed in amphibians, whilst allometric trajectories of the skull are relatively few and tend to be intraspecific or ontogenetic in focus. For example, Goldberg *et al*. (2024) assessed scaling of head width to snout–vent length (their proxy for body size) in six anuran species and found highly variable allometric patterns. The absence of interspecies and larger interclade allometric studies may reflect the assumption that the skull, neck, and associated skeletal morphology of amphibians are somewhat conserved (Handrigan & Wassersug, 2007). Studies of skull shape within amphibian subgroups, on the other hand, are commonplace (e.g. Trueb & Alberch, 1985; Ponssa & Candiot, 2012; Sherratt *et al*., 2014; Paluh, Stanley & Blackburn, 2020; dos Reis *et al*., 2021). Cranial width, in particular, is found to increase in relative terms as skulls become larger in anuran species (e.g. Bardua *et al*., 2021; Goldberg *et al*., 2024) and appears to be highly influenced by feeding function and microhabitat (Bardua *et al*., 2021). For example, relatively greater skull width appears to be associated with suction feeding in aquatic anurans and also facilitates a wider gape in predatory species (Bardua *et al*., 2021).

## III. NEW PERSPECTIVES ON TETRAPOD HEAD AND NECK ALLOMETRY AND ECOMORPHOLOGY

### (1) Aims

This review highlights several limitations, misconceptions and inconsistencies in existing studies of head and neck size in terrestrial tetrapods. To date a number of major groups, notably mammals, have received little to no attention, while studies that have examined allometric trends have done so using varied metrics for head, neck and overall body size. This disparity in metrics and analytical approaches means that it is currently difficult to reconcile allometric patterns and ecological adaptations across studies and subsequently across major taxonomic groups. Indeed, allometric patterns in some major groups, including mammals, have yet to be assessed. The goal of this study is therefore to examine head and neck evolution across major terrestrial tetrapod clades and dietary ecologies using a diverse range of metrics for quantifying the size of these body segments, and a single holistic measure of body size against which to judge allometric patterns. The specific aims of this study are: (*i*) to determine if different metrics for head and neck size yield disparate linear allometric signals across a large, taxonomically and ecologically diverse range of extinct and extant terrestrial tetrapods; (*ii*) to determine if allometric scaling in a range of metrics for head and neck size is better described by linear or quadratic models; (*iii*) to determine how major taxonomic groupings scale for a range of neck and head size metrics and if these metrics yield disparate allometric signals; and finally (*iv*) to determine how major trophic groupings scale for a range of neck and head size metrics and if these metrics yield disparate allometric signals.

In this study we therefore focus on broadening the taxonomic (and ecological) scope of previous allometric assessments of head and neck size and on evaluating the sensitivity of recovered allometric patterns to different head and neck size metrics. Our literature review highlighted additional limitations or challenges in the existing literature that are beyond the scope of systematic evaluation in the present study, notably the variable inclusion/exclusion of intervertebral discs in the measurement of neck length and highly varied metrics chosen to represent body size across previous studies. Here, rather than directly investigating the effects of these factors on allometric predictions, we standardise both parameters by measuring neck length as the sum of vertebral lengths (i.e. excluding the contribution of intervertebral discs) and using a single whole‐body metric to represent overall body size in our analyses.

### (2) Methodology

#### (a) Data set

Several head and neck shape and size metrics were quantified from 3D skeletal models of 116 amphibians, 47 birds, 55 non‐avian dinosaurs, 143 mammals, 46 extant non‐avian reptiles and three reptiliomorphs spread across the major groups within these clades, using the data set of Maher *et al*. (2022). Extant non‐avian reptiles and reptiliomorphs are hereafter grouped together under the term ‘other reptiles’ in our analysis for brevity. This data set consisted of 318 extant and 92 extinct species. The precise ontogenetic age and sex of many of the specimens was unknown. However, based on overall size, Maher *et al*. (2022) inferred that the majority of the extant specimens were sub‐adults or adults.

#### (b) Metrics for head and neck size

We calculated linear and volumetric measures of head and neck size for each specimen. For the linear analysis, two measures of head size (length and width) and one of neck size (length) were calculated. For the volumetric analysis, two different methods were used to calculate gross head and neck size: convex hulls (e.g. Sellers *et al*., 2012; Brassey & Sellers, 2014; Bates *et al*., 2016; Coatham *et al*., 2021; Macaulay *et al*., 2023) and α‐shapes (e.g. Brassey & Gardiner, 2015; Marek *et al*., 2021). Based on the broad consensus of previous studies, skull length was taken as the most posterior to the most anterior points, usually the distance between the occipital condyle and the anterior‐most projection of the skull, or to the end of the beak in birds (Fig. 1). Skull width was measured as the largest width of the skull, which tended to be the distance between the two zygomatic arches. The neck was considered as the skeletally defined cervical region, i.e. all vertebrae from the cranium to the first thoracic vertebra or rib (Romer, 1950). Neck length was calculated across this skeletal region by summing centrum lengths and did not include any estimates of size of intervertebral discs or cartilage.

**Fig. 3:**
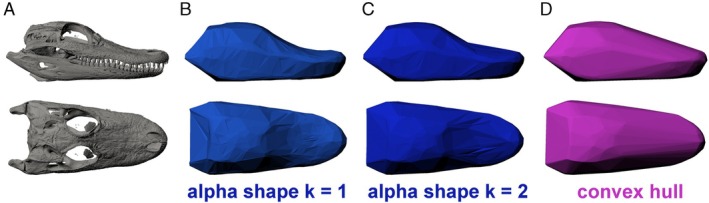
Volumetric representations of skull size in *Alligator mississippiensis* in lateral (top) and dorsal (bottom) view. (A) Computed tomography (CT) scan‐derived three‐dimensional model of the skull that was used to derive (B) a ‘finer’ fit of α‐shape when the α‐shape refinement coefficient *k* = 1.0, (C) a ‘coarser’ fit of α‐shape when *k* = 2.0, and (D) convex hull fit of α‐shape when α is infinite.

The α‐shape approach allows the investigator to set a parameter ‘*α*’ that determines the tightness of the volumetric fit, with large values producing coarse fits that are similar/identical to convex hulls and smaller values producing fits that ‘shrink‐wrap’ closer to the underlying point cloud (Gardiner, Behnsen & Brassey, 2018). By setting an appropriate value of *α*, the fit of the mathematically generated volumes is more anatomically realistic than simple convex hulls (Fig. 3). However, values of α that are too small will lead to fits that ‘break down’ and start to pass internally within the underlying point cloud, leading to fits that will underestimate volume and therefore mass (see Fig. 1 in Gardiner *et al*., 2018). Skull reconstructions were downsampled to 10,000 vertices where the original meshes exceeded this threshold value, to reduce the disparity between photogrammetric and computed tomography (CT) models. Two skulls had fewer than 1000 vertices in the data set and were subsequently removed from the analysis. To control for absolute changes in specimen size, the fit of the α‐shape is defined by a refinement coefficient *k* [see Gardiner *et al*. (2018) for details], which must be subjectively set by the investigator (*versus* no user‐defined subjectivity in the simpler convex hulling approach). To investigate the impact of this subjectivity on skull size patterns across the data set, three different α‐shape iterations were originally produced, where *k* was set to 0.5, 1 and 2 (Fig. 3). Visual inspection of these volumes revealed that the *k* = 0.5 iteration (the most refined or tightest‐fitting iteration) yielded poorly formed volumes in some models due to two distinct mechanisms. In skulls represented by particularly low resolution meshes (derived from photogrammetry or long‐range laser scans) the *k* = 0.5 α‐shapes often began to pass internally into the skulls and even break down into more than one mesh. Similarly undesirable results occurred in amphibians and particularly frogs, but here the mechanistic cause was skull shape rather scan resolution (most of our frog skeletal models are derived from microCT scans). Specifically, as α‐shapes became tighter and more refined, they began to sink into the orbits and develop a concave shape ventrally. In some instances, the *k* = 0.5 α‐shape volumes collapsed in on themselves. We therefore excluded *k* = 0.5 α‐shape iteration from our analyses.

As in Maher *et al*. (2022), whole‐body convex hull volume (WBCHV) was used as the measure of whole‐body size against which to judge allometric patterns. WBCHV is the sum of the minimum skeletal convex hull volume of all body segments in each 3D model. WBCHV is preferred as a proxy for overall body size because it utilises the entire skeleton rather than relying on a measure from a single body segment, which may bias further analyses due to potential allometric signals in that one body segment. Using WBCHV also allowed all linear and volumetric parameters to be assessed or normalised by the same size metric. Also, variability in scan/model resolution meant that popular alternative metrics (e.g. long bone circumference; Campione & Evans, 2012) could not be accurately and/or repeatably measured across the data set.

#### (c) Statistical analysis

To analyse trends in the skull and neck proportions using phylogenetically informed statistics, we used the existing tree accompanying this data set from Maher *et al*. (2022). Branch lengths were calculated by time‐calibrating the tree using first and last occurrences of each species from the *Palaeobiological* database (www.paleobiodb.org) and *Fossilworks* (www.fossilworks.org). These data were then combined with the phylogenetic tree in R v4.1.2 (R Core Team, 2021) using the package *strap* (Bell & Lloyd, 2015) to produce a time‐calibrated tree. To examine variations and correlations in body shape and ecological variables, taxa were classified into dietary categories based on the current consensus in the literature following Maher *et al*. (2022). Specifically, the dietary type of each species was classified as either carnivore (*N* = 124), herbivore (*N* = 120), insectivore (*N* = 94), omnivore (*N* = 64) or piscivore (*N* = 8).

To examine how each skull and neck metric scaled with body size (WBCHV) across the data set, a regression analysis using phylogenetic generalised least squares (PGLS) in the R package *caper* was conducted (Freckleton, 2002). This approach follows a generalised linear model, calculating the slope, intercept, confidence and prediction intervals, and adjusting the expected covariance according to phylogenetic signal (Symonds & Blomberg, 2014). Quadratic models were also tested so see if they provided a statistically better fit to scaling trends than linear fits in the log_10_ parameter *versus* body mass data set (Campione, 2017), as in Maher *et al*. (2022). Sauropod dinosaurs represent by far the largest animals in our data set and are characterised by relatively long necks but small heads. To examine their impact on allometric trends in tetrapods as a whole we conducted additional ordinary least squares (OLS) regressions with and without sauropods excluded from the whole data set.

All analysis was carried out in R using the packages *qpcr*, *ape*, *Geiger* and *nlme* (Andrej‐Nikolai, 2014; Pennell *et al*., 2014; Campione, 2017; Pinheiro, Bates & R Core Team, 2025). A statistically significant second‐degree coefficient established the nonlinear nature of the data if present. Models were compared using Akaike information criteria for limited sample sizes (AICc) and standard errors of the estimate. Pagel's lambda (*λ*) (Pagel, 1999), was used to estimate the strength of the phylogenetic signal in the analyses. Phylogenetic analysis of covariance (phylANCOVA) was used to test for differences in the allometric relationships and body shape between dietary groups using the approach of Smaers & Rohlf (2016) implemented in the R package *evomap* (Smaers & Mongle, 2014). Here, because our specific goal is to assess differential changes in head and neck size across taxonomic and ecological groupings, we focus on significant (*P* < 0.05) differences in slope values and values for slope when the intercept is considered. For the purpose of comparing how different linear and volumetric metrics influence scaling patterns in tetrapod heads and necks, we categorised slopes between 0.321 and 0.339 for a linear metric, and between 0.98 and 1.02 for a volume metric as ‘isometric’ unless their confidence intervals exclude isometry. Where slopes fall outside these ranges qualitative trends emerging from these models are described as showing definitive positive or negative allometry only if the 95% confidence intervals of the slope exclude isometry. Where 95% confidence intervals for the slope include isometry, we note this distinction in our narrative text and use the designations ‘*~negative*’ and ‘~*positive*’ in our summary tables.

## IV. RESULTS

### (1) Allometric patterns for different head and neck metrics across terrestrial tetrapods

**Fig. 4:**
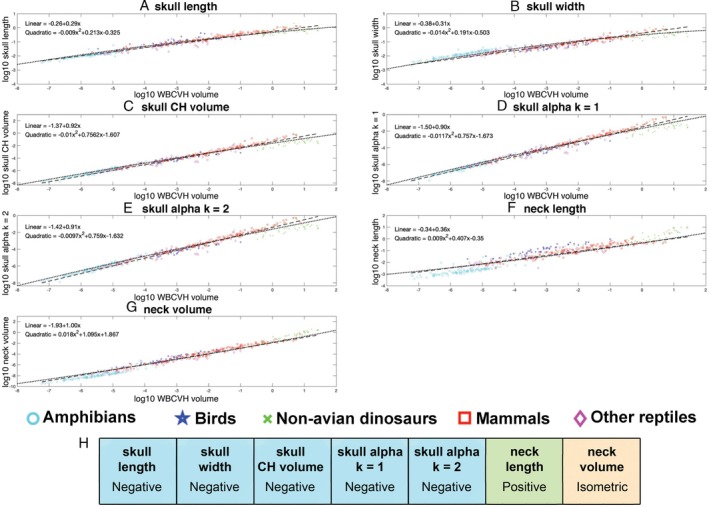
Head and neck size allometry in terrestrial tetrapods. Scaling relationships between different metrics for head and neck segment size and overall body size (whole body convex hull volume) for 410 terrestrial tetrapods using phylogenetically informed linear (thick dashed lines) and quadratic (thin dotted lines) fits. The head is represented by (A) skull length, (B) skull width, (C) head convex hull (CH) volume and two α‐shape volumes where the α‐shape refinement coefficient (*k*) was (D) 1 and (E) 2. The neck is represented by (F) neck length and (G) convex hull volume. Where the head and neck are represented by linear metrics, isometry would result in a slope of 0.33, while for volumetric metrics isometry would result in a slope of 1. (H) Summary of the qualitative allometric patterns for each metric. Full details of the regression model information can be found in Table S1. CHV, convex hull volume.

Linear allometric relationships for all variables were statistically significant (Fig. 4; see online Supporting Information, Table S1). All linear and volumetric metrics for skull size scaled with negative allometry (skull length: slope = 0.29 ± 0.01; skull width: slope = 0.31 ± 0.01; skull convex hull volume: slope = 0.92 ± 0.035; skull volume measured by α‐shape methods: slopes = 0.9–0.91 ± 0.02; Table S1; Fig. 4A–E). Therefore, all metrics suggest a relative decrease in head size as overall body size increases in terrestrial tetrapods. Exclusion of sauropod dinosaurs from OLS regression models of the whole data set increased slopes across all parameters (i.e. reduced the magnitude of negative allometry), but in no instances were these changes so large that isometry is included in the 95% confidence intervals (Table S2).

By contrast, different metrics yielded disparate allometric relationships for the neck. Neck length scaled with positive allometry (slope = 0.36 ± 0.02; Table S1; Fig. 4F), while neck volume scaled isometrically with body size (slope = 1.00 ± 0.025; Table S1; Fig. 4G). Therefore, these metrics yield different qualitative patterns in neck size scaling across terrestrial tetrapods as a whole; necks become longer as overall body size increases, while neck volume remains a relatively constant proportion of overall skeletal volume at all body sizes. Exclusion of sauropod dinosaurs from OLS regression models of the whole data set reduced the slopes for both neck length and volume (Table S2), but the qualitative scaling patterns were unaffected.

**Fig. 5:**
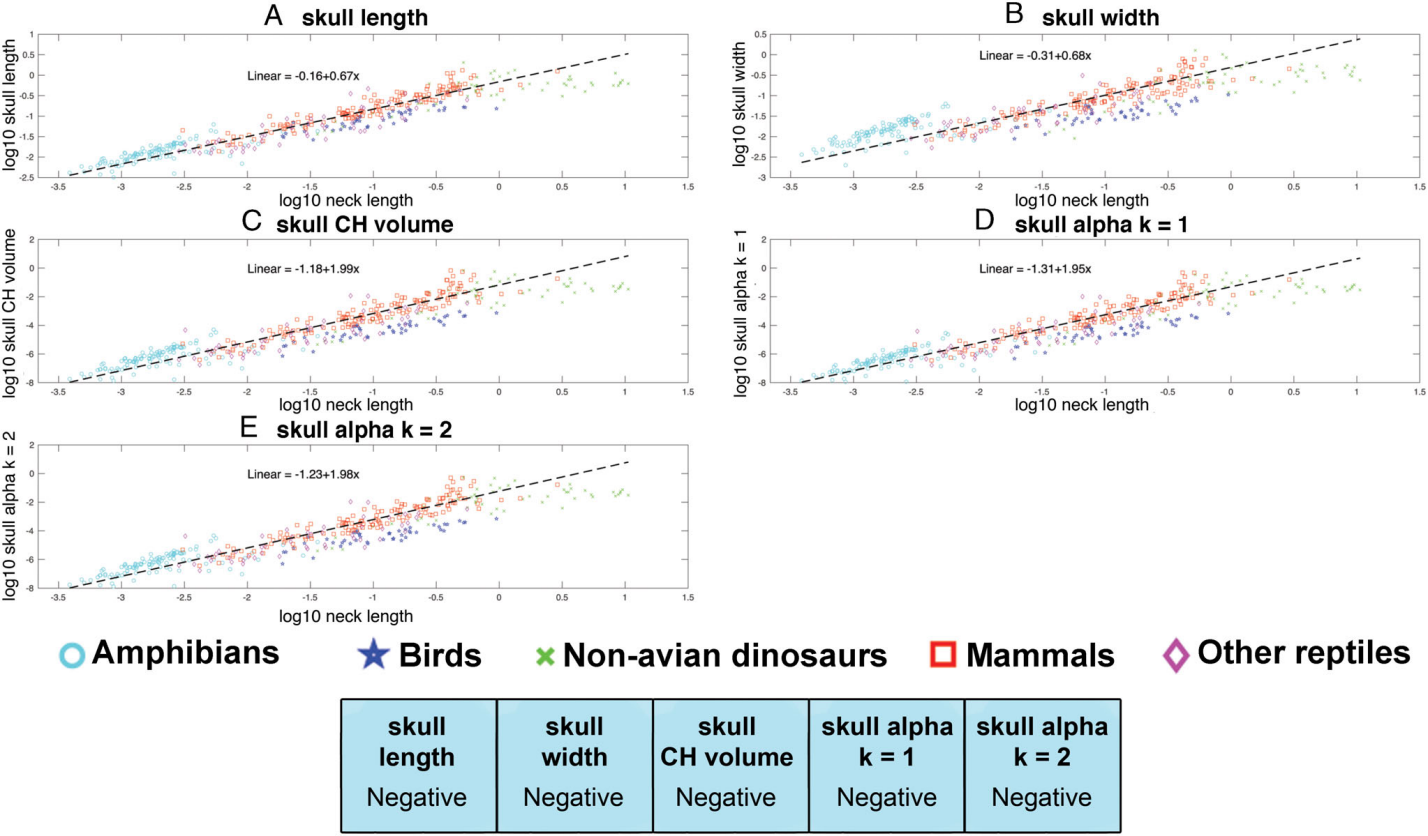
Scaling relationships between different metrics for head size *versus* neck length in 410 terrestrial tetrapods using phylogenetically informed linear fits. The head is represented by (A) skull length, (B) skull width, (C) head convex hull (CH) volume and three α‐shape volumes where the α‐shape refinement coefficient (*k*) was (D) 1 and (E) 2. Where the head is represented by linear metrics, isometry would result in a slope of 1, while for volumetric metrics isometry would result in a slope of 3. (F) Summary of the qualitative allometric patterns for each metric. Full details of the regression model information can be found in Table S3. CHV, convex hull volume.

Skull length (slope = 0.67) and width (slope = 0.68) scaled with negative allometry relative to neck length (with 95% confidence intervals below isometry, where in this case isometry would result in a slope of 1), indicating that on average skull size decreases relative to neck length in tetrapods according to both these metrics (Fig. 5; Table S3). All three volumetric measures of skull size also scaled with negative allometry (slopes = 1.95–1.99; Fig. 5; Table S3), with 95% confidence intervals below isometry (expected slope for isometry = 3). Exclusion of sauropod dinosaurs from OLS regression models of the whole data set increased slopes across all parameters (i.e. reduced the magnitude of negative allometry), but in no instances were these changes sufficient to include isometry in the 95% confidence intervals (Table S4).

### (2) Differential scaling in head and neck size in small‐ *versus* large‐bodied terrestrial tetrapods

Second‐degree coefficients for phylogenetically informed quadratic models fitted through all linear and volumetric head and neck measurements were examined (Table S5; Fig. 4). Phylogenetically informed quadratic models for all parameters except skull length were statistically significant (Table S5), with skull length falling only slightly above significance (*P* = 0.0532; Table S5). AICc values were slightly lower for linear fits for the linear segment size metrics (skull width and neck length) and skull convex hull volume, but marginally lower for quadratic models for neck convex hull volume and the two skull α‐shape volumes, although these values were relatively similar in all cases. Therefore, allometric patterns in linear size metrics are slightly better described by a simple linear model, while a non‐linear quadratic fit better describes the scaling of volumetric size metrics (Fig. 4).

**Fig. 6:**
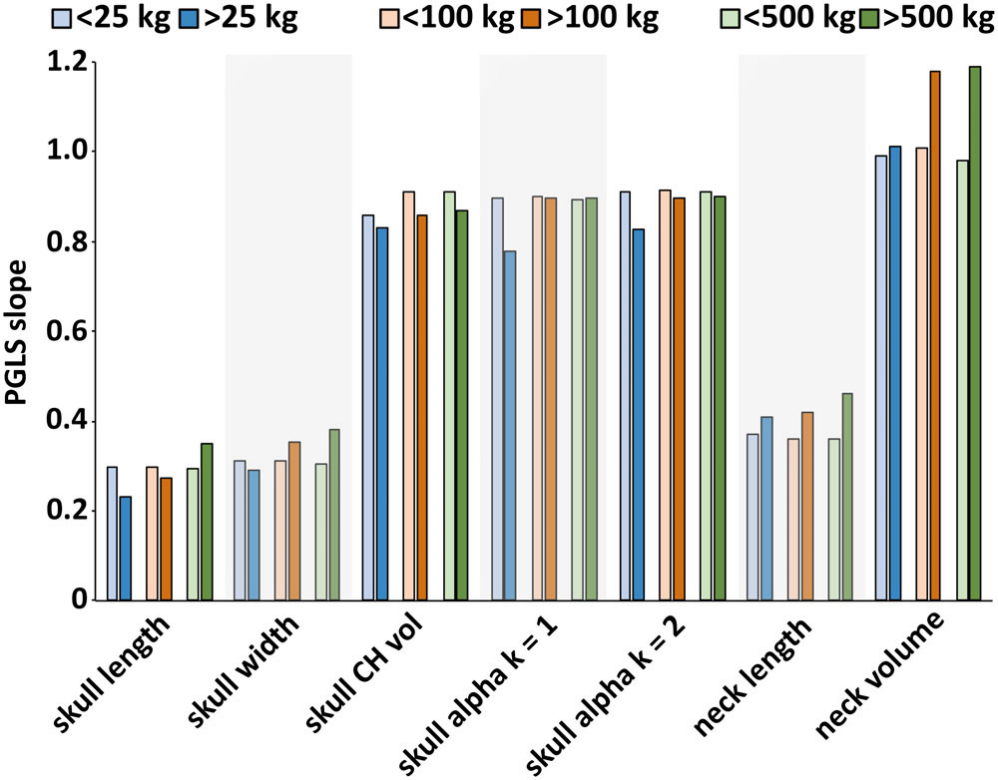
Assessing differential scaling of the head and neck in small‐ to large‐sized tetrapods. Phylogenetic generalised least squares (PGLS) regression slopes for different head and neck size metrics with taxa split into body size bins (<25 kg *versus* > 25 kg; <100 kg *versus* > 100 kg; <500 kg *versus* > 500 kg). Skull alpha *k* = 1 and skull alpha *k* = 2 refer to the volumetric iterations where the α‐shape refinement coefficient (*k*) was 1 and 2, respectively. CH vol, convex hull volume.

To examine the nature of this non‐linearity on head and neck segment allometry, the slopes of a series of size thresholds (or bins) within the full data set were compared (Fig. 6). For skull convex hull volume greater negative allometry was always recovered in the larger size group regardless of whether the size threshold was drawn at 25 kg, 100 kg or 500 kg (Tables S6–S8; Fig. 6). For α‐shape skull volumes 1 (*k* = 1.0) and 2 (*k* = 2.0), greater negative allometry was recovered in taxa above 25 kg *versus* below 25 kg. However, when taxa were split at 100 kg or 500 kg the magnitudes of negative allometry were similar (Tables S6–S8; Fig. 6). For neck volume, taxa above and below 25 kg both scaled close to isometry. However, when taxa were split at larger body sizes, the >100 kg and > 500 kg taxa showed strong positive allometry, while the smaller size‐bins remained isometric (Tables S6–S8; Fig. 6).

### (3) Taxonomic patterns in head and neck size

#### (a) Head size

**Fig. 7:**
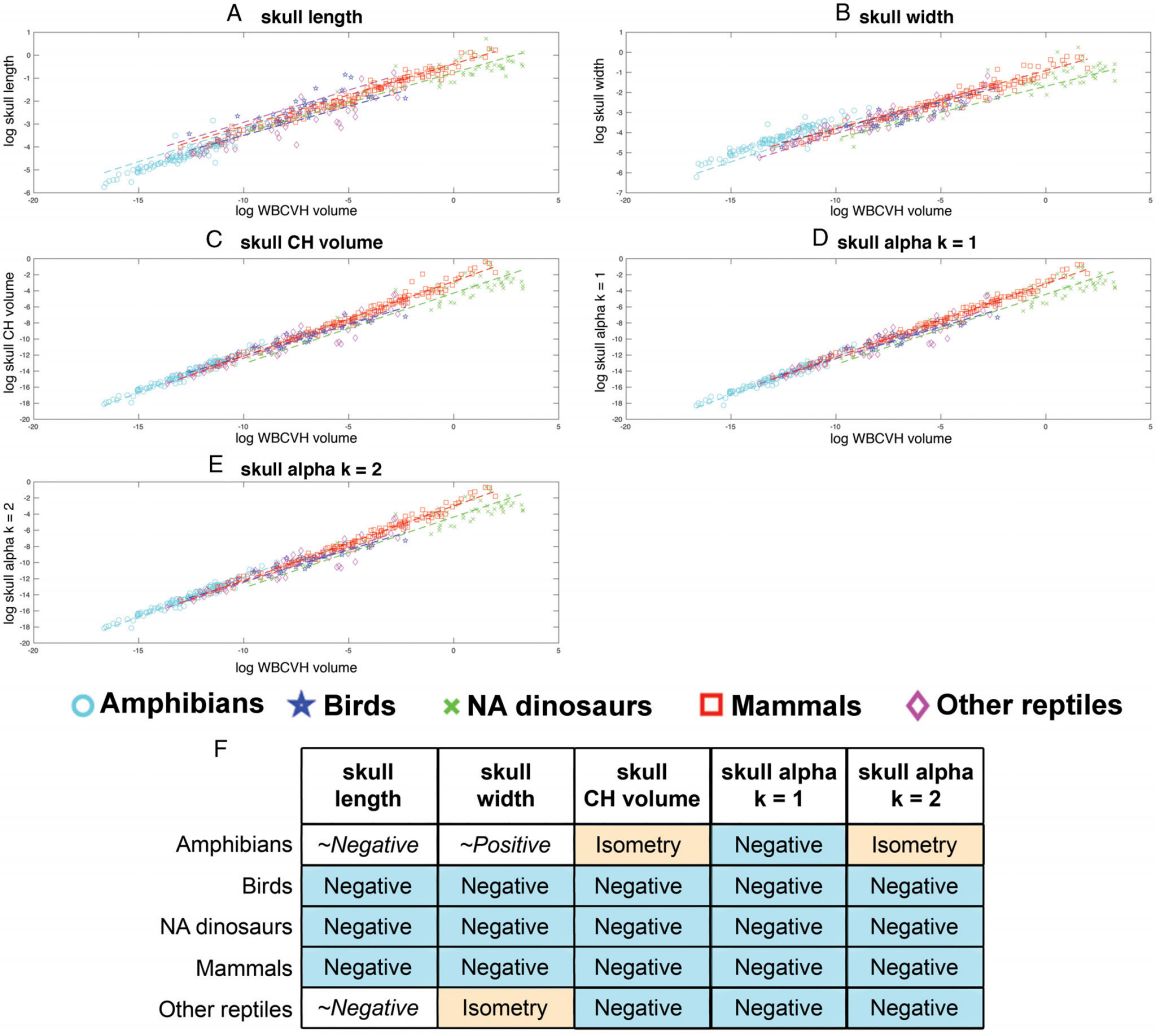
Head allometry in different tetrapod groups. Scaling relationships between different metrics for head size and overall body size (whole body convex hull volume) in major tetrapod clades using phylogenetically informed linear fits. The head is represented by (A) skull length, (B) skull width, (C) head convex hull (CH) volume and three α‐shape volumes where the α‐shape refinement coefficient (*k*) was (D) 1 and (E) 2. Where the head is represented by a linear metric, isometry would result in a slope of 0.33, while for volumetric metrics isometry would result in a slope of 1. (F) Summary of the qualitative allometric patterns for each metric, where negative allometry with isometry not included in the 95% confidence intervals is colour‐coded blue, and white cells indicate negative or positive allometric slopes but with isometry included within the 95% confidence intervals. The data set consisted of 116 amphibians, 47 birds, 55 non‐avian (NA) dinosaurs, 143 mammals, and 49 other reptiles (46 extant non‐avian reptiles, and 3 reptiliomorphs). Full details of the regression model information can be found in Table S9. CHV, convex hull volume.

Linear allometric relationships for all head variables in all major taxonomic subgroups were statistically significant (Table S9). Skull length showed negative allometry in all taxonomic groups, ranging from a slope of 0.254 in birds to 0.294 in amphibians. Only the 95% confidence intervals of the slopes for amphibians and other reptiles included isometry (Table S9). The slopes of birds and amphibians were not recovered as statistically different (Table S10). However, amphibians were statistically closer to isometry than non‐avian dinosaurs, mammals, and other reptiles when the contribution of their intercepts was considered, but not in their raw slope values alone (Table S10; Fig. 7A). Birds showed statistically greater negative allometry than non‐avian dinosaurs, mammals and other reptiles both with and without inclusion of their intercept values (Table S10; Fig. 7A).

Taxonomic groups were qualitatively more divergent in skull width allometry (Fig. 7B; Table S9). Amphibians scaled with positive allometry (slope = 0.351), although negative allometry is narrowly included within the 95% confidence intervals. Other reptiles scaled with isometry (slope = 0.33), while mammals (slope = 0.289), birds (slope = 0.265) and non‐avian dinosaurs (slope = 0.26) scaled with negative allometry (Fig. 7B; Table S9). PhylANCOVA tests showed that non‐avian dinosaurs were not statistically different to mammals or other reptiles in skull width, and that mammals and other reptiles were not statistically different (Table S11). However, positive allometry in amphibians was statistically different to the isometry and negative allometry seen in the other groups, but in the case of birds a significant difference was only recovered for amphibians when the contribution of their intercepts was considered (Table S11). Birds scaled with significantly greater negative allometry than other reptiles and mammals in skull width with and without consideration of the contribution of their intercepts, while non‐avian dinosaurs showed statistically greater negative allometry than birds but only when differences in their intercepts were not considered (Table S11).

Skull convex hull volume scaled negatively for all groups (slopes = 0.788–0.931), with upper 95% confidence below isometry, except amphibians (slope = 0.987) which scaled isometrically (Table S9; Fig. 7C). Mammals were not statistically different from birds, non‐avian dinosaurs and other reptiles in skull convex hull volume (Table S12). Non‐avian dinosaur and other reptiles slopes were not significantly different, but were recovered as significantly different when the contribution of their intercepts was also considered (Table S12). Isometric scaling in amphibians was statistically different to negative allometry in all other groups when the contribution of differing intercepts was accounted for (Table S12). However, amphibians were recovered as statistically different to mammals with and without consideration of the intercept (Table S12).

The two α‐shape iterations showed the same relative allometric pattern in each taxonomic group: as *k* increases, the slopes of each taxonomic group increased and moved slightly closer to isometry (Table S9; Fig. 7D, E). By our qualitative categorisation, amphibians show isometry when α‐shape *k* = 2 (slope 0.984) and negative allometry when α‐shape *k* = 1 (slope = 0.952). These slopes were higher than other taxonomic groups, with birds (slopes = 0.786 and 0.785, respectively) showing the strongest negative allometry (Fig. 7D, E; Table S9). In both α‐shape iterations, mammals were not statistically different to non‐avian dinosaurs and other reptiles, while non‐avian dinosaurs only differed statistically in their slope to other reptiles in the α‐shape volume iteration *k* = 2 when the contribution of the intercept was considered (Tables S13 and S14). Across both α‐volume iterations, amphibians were recovered with a statistically higher slope than all other groups with and without the contribution of the intercept (Tables S13 and S14). Birds had a significantly lower slope than non‐avian dinosaurs in the α‐shape volume iteration *k* = 2, but only when the contribution of the intercept was considered (Table S14). This qualitative difference was recovered as marginally non‐significant (*P* = 0.054) in the α‐shape volume iteration *k* = 1 (Table S13). Birds were not recovered as significantly different to mammals or other reptiles in either α‐shape volume iterations (Tables S13 and S14).

Overall, therefore, the different head metrics mostly yielded the same qualitative picture of scaling patterns within and across major taxonomic groups (Fig. 7F). Both linear measures of head size (skull length and width) suggested negative allometry in birds, non‐avian dinosaurs and mammals, with 95% confidence intervals below isometry. In other reptiles, skull length scaled with negative allometry (but with 95% confidence intervals including isometry), while skull width scaled isometrically and all three volumetric metrics scaled with negative allometry. Skull length scaled with negative allometry in amphibians, but with 95% confidence intervals inclusive of isometry, while by contrast skull width scaled with positive allometry in this group with the lower 95% confidence interval narrowly including isometry. The same qualitative (negative) allometric patterns were recovered in all volumetric measures of head size for birds, non‐avian dinosaurs and mammals. In amphibians, convex hull volume and α‐shape *k* = 2 iterations both scaled isometrically, while the α‐shape *k* = 1 models scaled with negative allometry, contrasting with the positive allometry seen in skull width.

#### (b) Neck size

**Fig. 8:**
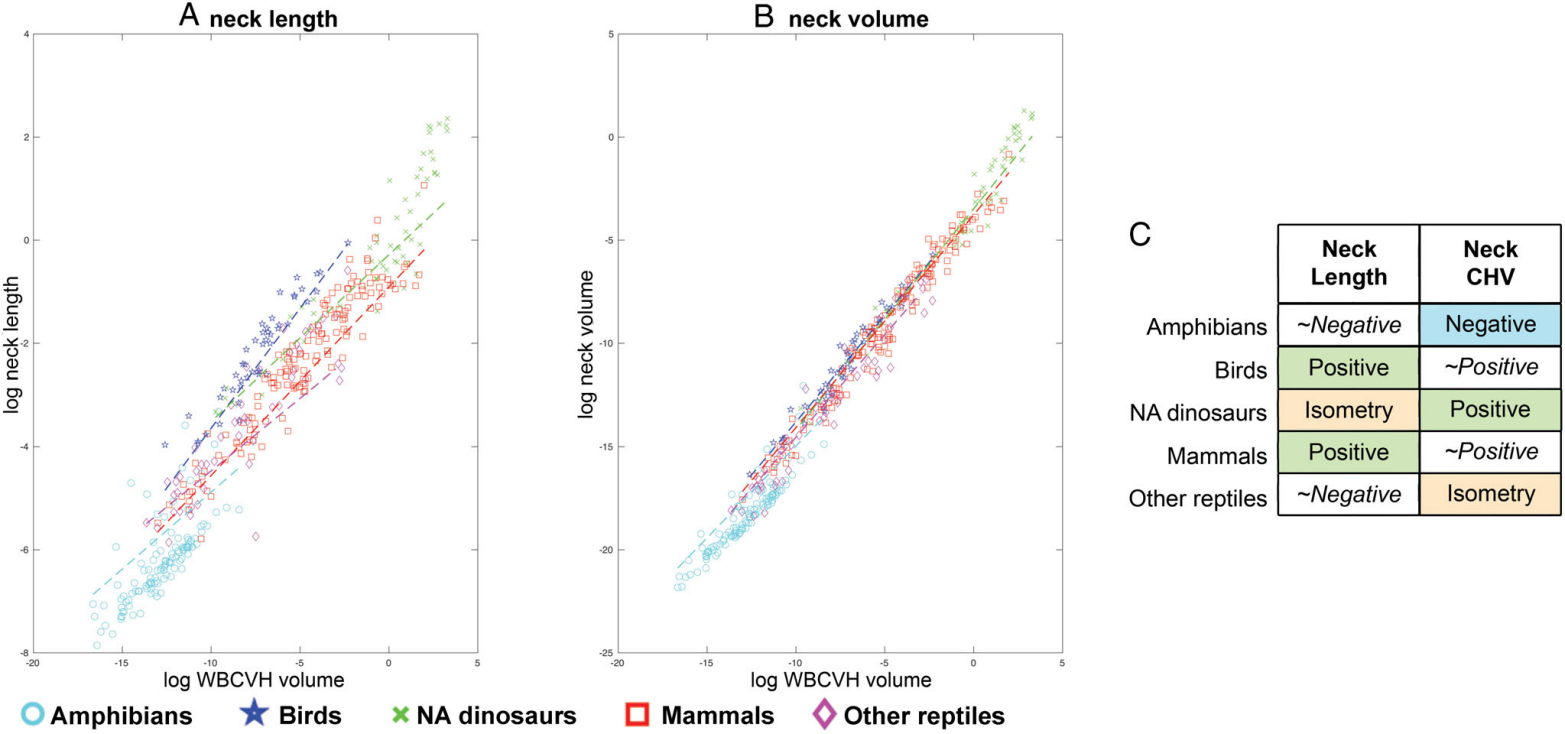
Neck allometry in different tetrapod groups. Scaling relationships between different metrics for neck segment size and overall body size (whole body convex hull volume) in major tetrapod clades using phylogenetically informed linear fits. The neck is represented by overall (A) length and (B) convex hull volume. A slope of 0.33 would represent isometry in neck length, while for neck volume isometry would result in a slope of 1. (C) Summary of the qualitative allometric patterns for each metric, where positive and negative allometry with isometry not included in the 95% confidence intervals is colour‐coded green and blue respectively, and white cells indicate negative or positive allometric slopes but with isometry included within the 95% confidence intervals. The data set consisted of 116 amphibians, 47 birds, 55 non‐avian (NA) dinosaurs, 143 mammals, and 49 other reptiles (46 extant non‐avian reptiles, and 3 reptiliomorphs). Full details of the regression model information can be found in Table S9. CHV, convex hull volume.

Taxonomic groups were highly disparate in their neck length allometries (Fig. 8A; Table S9). Birds scaled with strong positive allometry (slope = 0.464) and mammals with more modest positive allometry (slope = 0.365) (Table S9). Neck length in non‐avian dinosaurs scaled with isometry (slope = 0.323), with very broad 95% confidence intervals (Fig. 8A; Table S9), while amphibians (slope = 0.299) and other reptiles (slope = 0.281) scaled with stronger negative allometry but also with isometry included within the 95% confidence intervals (Fig. 8A; Table S9). Non‐avian dinosaurs were not statistically different to mammals or other reptiles in neck length allometry, and mammals and other reptiles were also not statistically different (Table S15). Amphibians were not significantly different to birds, but were statistically different to mammals, non‐avian dinosaurs and other reptiles in their slopes when the contribution of their intercepts was considered (Table S15). Birds were not statistically different to mammals and other reptiles but did scale with significantly greater positive allometry than non‐avian dinosaurs but only when the contribution of their intercepts was not considered (Fig. 8A; Table S15).

Neck volume scaled slightly above isometry in mammals and birds (slopes = 1.03), but in both cases isometry was included within 95% confidence intervals. Non‐avian dinosaurs scaled with positive allometry (slope = 1.07), with lower 95% confidence intervals narrowly above isometry (Table S9). Negative allometry was recovered in amphibians (slope = 0.89) and other reptiles scaled isometrically (slope = 0.993) (Fig. 8B; Table S9). Other reptiles were not statistically different to mammals or non‐avian dinosaurs in neck volume allometry (Table S16). Amphibians were not statistically different to non‐avian dinosaurs, but showed significantly greater negative allometry than birds, mammals and other reptiles but only when the contribution of intercepts was considered (Fig. 8B; Table S16). Non‐avian dinosaurs were recovered with a significantly greater slope than birds, but only differed statistically to mammals when the contribution of intercepts was considered (Table S16).

The choice of metric (length *versus* volume) to represent neck size therefore did have qualitative impacts on the allometric patterns for most taxonomic groups. Clear positive allometry was recovered birds and mammals in neck length, but isometry cannot be excluded for these groups when the neck is represented by volume (Fig. 8C; Table S9). Amphibians scale with unequivocal negative allometry in neck volume, but isometry is included within confidence intervals for neck length. Isometry was recovered for other reptiles in neck volume, while neck length had a negative slope in this group but with confidence intervals inclusive of isometry. Non‐avian dinosaurs were recovered as isometric in their neck length, but scaled with positive allometry in neck volume (Fig. 8C; Table S9).

#### (c) Head versus neck scaling

**Fig. 9:**
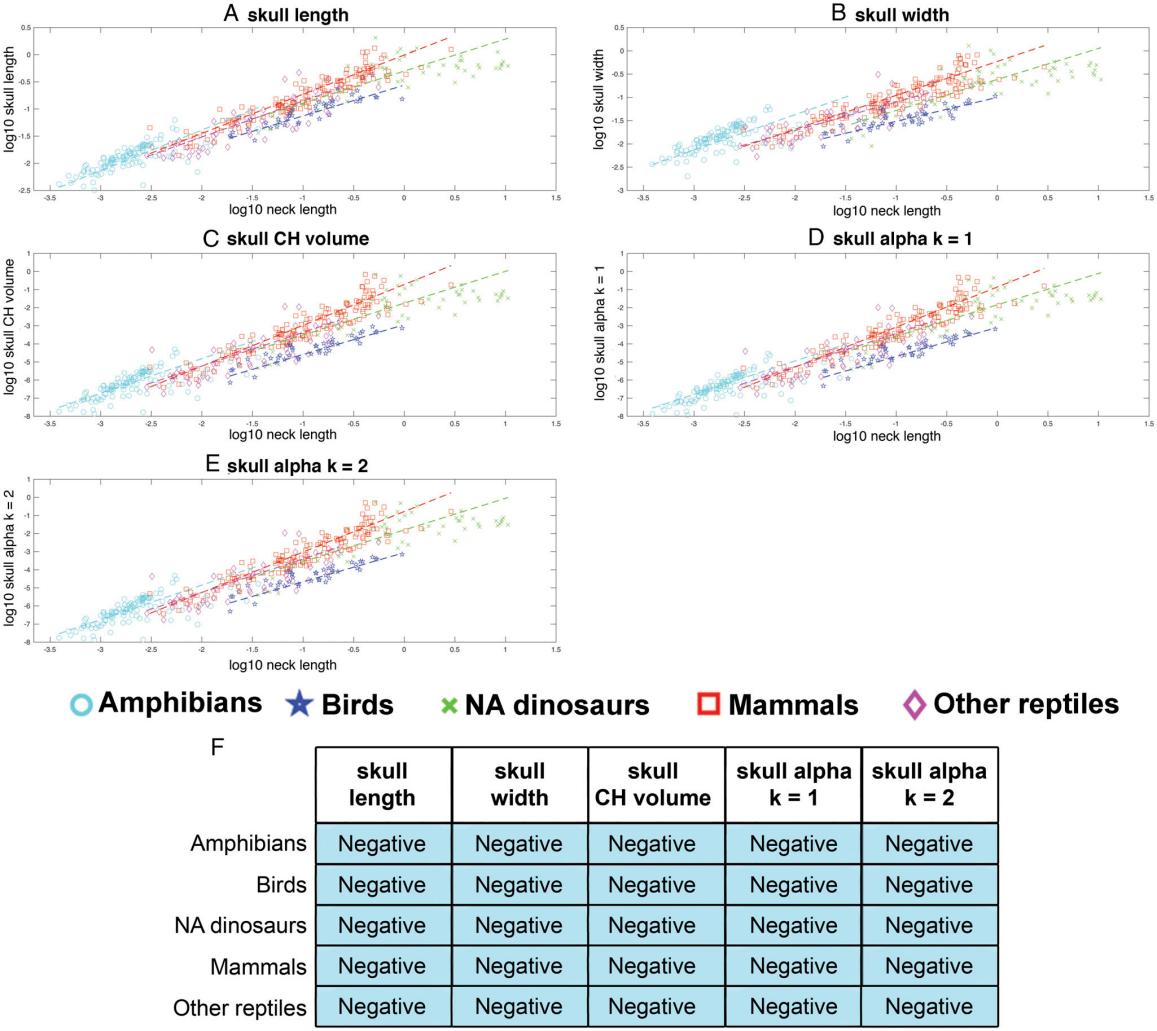
Head–neck scaling in different tetrapod groups. Scaling relationships between different metrics for head size and neck length in major tetrapod clades using phylogenetically informed linear fits. The head is represented by (A) skull length, (B) skull width, (C) head convex hull (CH) volume and three α‐shape volumes where the α‐shape refinement coefficient (*k*) was (D) 1 and (E) 2. Where the head is represented by a linear metric, isometry would result in a slope of 1, while for volumetric metrics isometry would result in a slope of 3. (F) Summary of the qualitative allometric patterns for each metric, where negative allometry with isometry not included in the 95% confidence intervals is colour‐coded blue. The data set consisted of 116 amphibians, 47 birds, 55 non‐avian (NA) dinosaurs, 143 mammals, and 49 other reptiles (46 extant non‐avian reptiles, and 3 reptiliomorphs). Full details of the regression model information can be found in Table S9. CHV, convex hull volume.

Linear relationships between neck length and skull size metrics were statistically significant (*P* < 0.05) for all taxonomic groups (Fig. 9; Table S17). All skull metrics scaled with negative allometry relative to neck length in all taxonomic groups, indicating that on average skull size decreases relative to neck length in all cases (Fig. 9; Table S17). Birds scaled with the strongest negative allometry in all linear and volumetric measures of head size and were generally significantly different to other groups except dinosaurs (Tables S18–S22). Amphibians scaled closest to isometry in the linear metrics and were significantly different to all other taxonomic groups, while mammals were closest to isometry in all volumetric metrics for head size and again were largely significantly different to all other groups in these metrics (Tables S18–S22).

### (4) Trophic patterns in head and neck size

#### (a) Head size

**Fig. 10:**
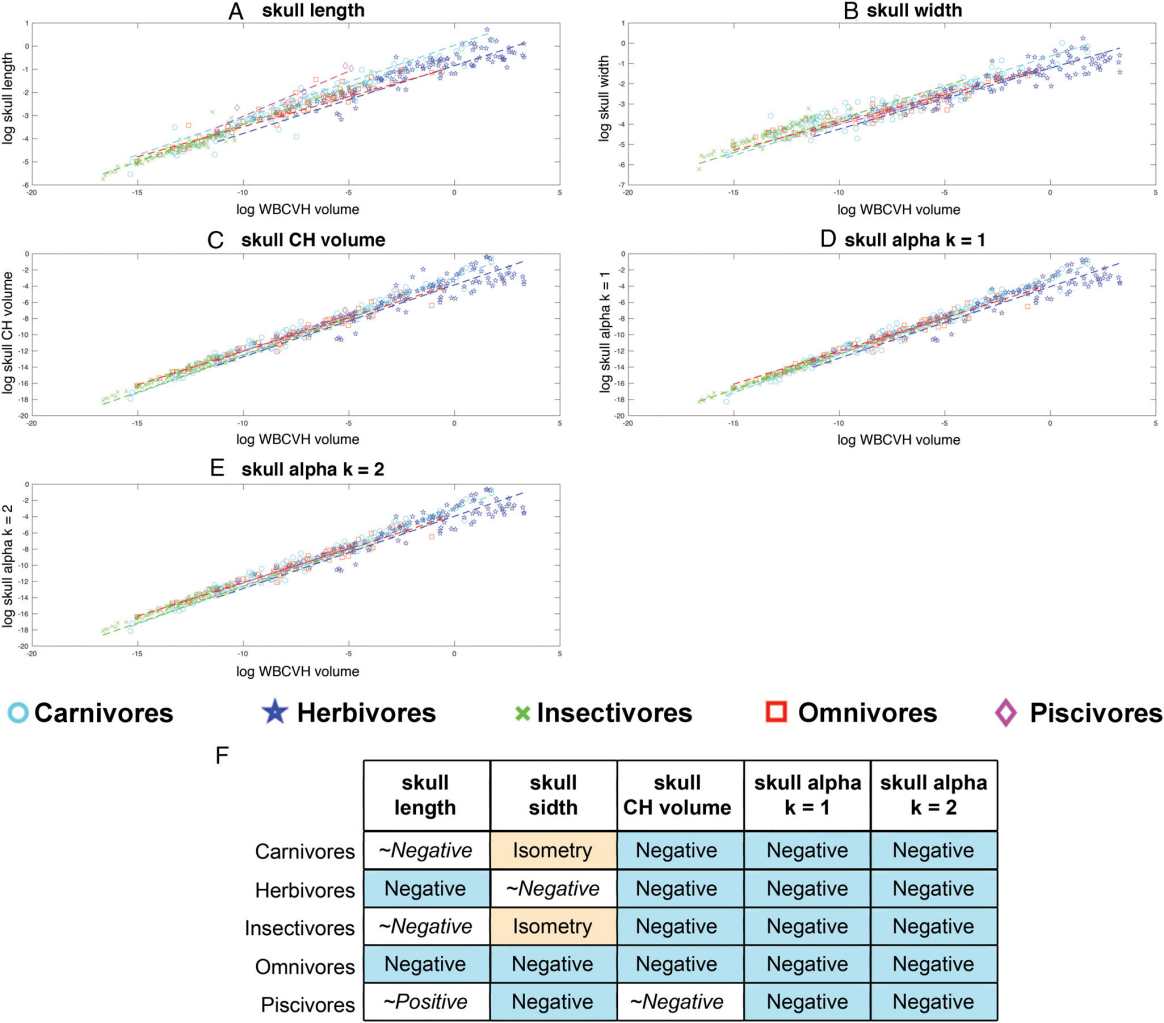
Head allometry in different dietary groups. Scaling relationships between different metrics for head size and overall body size (whole body convex hull volume) in major tetrapod trophic groups using phylogenetically informed linear fits. The head is represented by (A) skull length, (B) skull width, (C) head convex hull (CH) volume and three α‐shape volumes where the α‐shape refinement coefficient (*k*) was (D) 1 and (E) 2. Where the head is represented by a linear metric, isometry would result in a slope of 0.33, while for volumetric metrics isometry would result in a slope of 1. (F) Summary of the qualitative allometric patterns for each metric, where negative allometry with isometry not included in the 95% confidence intervals is colour‐coded blue, and white cells indicate negative or positive allometric slopes but with isometry included within the 95% confidence intervals. The data set consisted of 124 carnivores, 120 herbivores, 94 insectivores, 64 omnivores and 8 piscivores. Full details of the regression model information can be found in Table S23. CHV, convex hull volume.

Linear allometric relationships for all variables were statistically significant (Table S23). Skull length showed negative allometry in carnivores (slope = 0.316) and insectivores (slope = 0.322), and positive allometry in piscivores (slope = 0.389), but in all three cases 95% confidence intervals included isometry (Fig. 10A; Table S23). Herbivores (slope = 0.293) and omnivores (slope = 0.259) scaled with negative allometry (Fig. 10A; Table S23). In terms of skull length, herbivores were recovered as universally different to all other trophic groups, but carnivores differed statistically from all other (non‐herbivore) trophic groupings only when the contribution of their intercepts was considered (Table S24). Insectivores, piscivores and omnivores were not significantly different to each other (Table S24).

For skull width, carnivores (slope = 0.327) and insectivores (slope = 0.33) scaled isometrically, whilst omnivores (slope = 0.274) and piscivores (slope = 0.265) scaled with negative allometry (Fig. 10B; Table S23). Herbivores (slope = 0.301) also scaled with negative allometry, but with upper confidence intervals narrowly including isometry. Carnivores were recovered as significantly different to all other trophic groupings (Table S25), while herbivores were statistically different to omnivores, piscivores and insectivores, but the in latter case only when the contribution of intercepts was not considered (Table S25). Insectivores, piscivores and omnivores were not significantly different to each other (Table S25).

Skull convex hull volume scaled with negative allometry for all trophic groupings, ranging from a slope of 0.831 in omnivores to 0.954 in carnivores (Fig. 10C; Table S23). However, the upper 95% confidence interval for piscivores included isometry. Carnivores were significantly different to all other trophic groupings, although were only recovered as different to herbivores when the contribution of intercepts was not considered (Table S26). Herbivores were found to be significantly different to insectivores and omnivores, but their slopes were only statistically different to piscivores when the contribution of intercepts was included (Table S26). Omnivores and piscivores were not statistically different from each other, while insectivores were significantly different from both these groups when the contribution of intercepts was not considered (Table S26).

Both α‐shape iterations showed negative allometry and the same relative pattern within each trophic group (Fig. 10D, E). That is, as *k* increased the slope of each trophic group increased and moved closer to isometry (Fig. 10D, E; Table S23). Carnivores showed the least‐marked negative allometry (slopes = 0.940 and 0.948), while omnivores showed the most marked negative allometry (slopes = 0.820 and 0.832) (Fig. 10D, E; Tables S23, S27 and S28). In both α‐shape iterations carnivores and herbivores were statistically different from all other trophic groupings, and insectivores, omnivores and piscivores were not statistically different to one another (Tables S27 and S28).

Therefore, only in omnivores do the different head size measures unequivocally deliver the same qualitative allometric pattern across trophic groups (i.e. negative allometry, without isometry included in the 95% confidence intervals; Fig. 10F). All linear and volumetric metrics suggested negative allometry in herbivores, although for skull width the upper 95% confidence interval included isometry. Carnivores and insectivores were recovered with unequivocal negative allometry in all volumetric measures, but isometric scaling for skull width, while the negative allometric slope for skull length included isometry in its 95% confidence intervals. In piscivores, the two α‐shape iterations and skull width suggested negative allometry, while the negative allometric slope for skull convex hull volume and positive allometry slope for skull length both included isometry in their 95% confidence intervals (Fig. 10F).

#### (b) Neck size

**Fig. 11:**
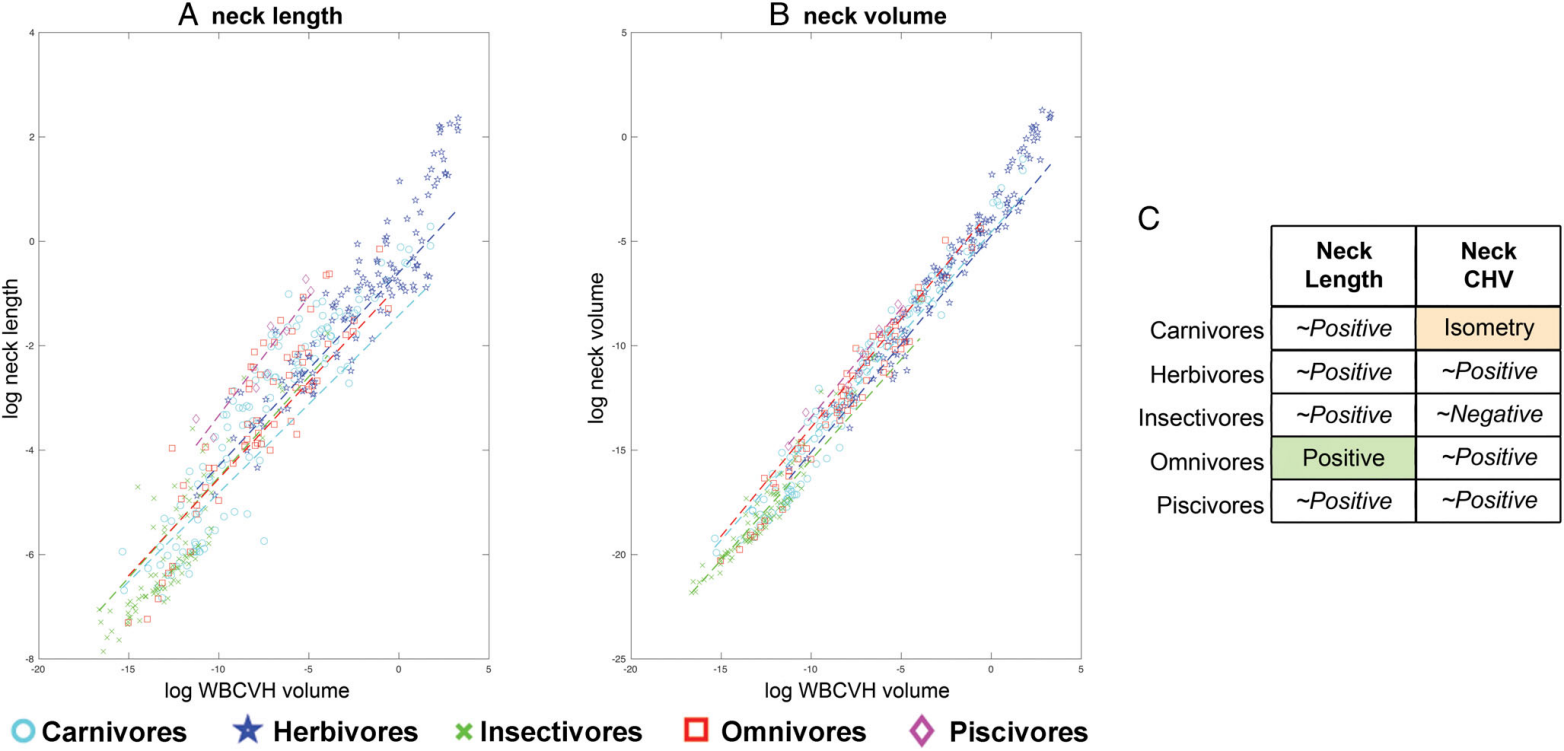
Neck allometry in different dietary groups. Scaling relationships between different metrics for neck segment size and overall body size (whole body convex hull volume) in major tetrapod trophic groups using phylogenetically informed linear fits. The neck is represented by overall (A) length and (B) convex hull volume. (C) Summary of the qualitative allometric patterns for each metric, where positive allometry with isometry not included in the 95% confidence intervals is colour‐coded green, and white cells indicate negative or positive allometric slopes but with isometry included within the 95% confidence intervals. A slope of 0.33 would represent isometry in neck length, while for neck volume isometry would be a slope of 1. The data set consists of 124 carnivores, 120 herbivores, 94 insectivores, 64 omnivores and 8 piscivores. Full details of the regression model information can be found in Table S23. CHV, convex hull volume.

Neck length scaled with positive allometry across all trophic groupings (Fig. 11A; Table S23). However, with the exception of omnivores, the 95% confidence intervals included isometry. Piscivores scaled with the highest positive allometry (slope = 0.455) and carnivores with the lowest positive allometry (slope = 0.340). Carnivores had significantly lower slopes than herbivores (slope = 0.370) when the contribution of intercepts was not considered, but conversely only differed statistically from other groups when differences in intercepts were considered (Table S29). Herbivores were not significantly different to omnivores or piscivores but were recovered as statistically different to insectivores when the contribution of intercepts was not considered (Table S29). Insectivores, piscivores and omnivores were not significantly different to each other in neck length allometry (Table S29).

Neck convex hull volume scaled with positive allometry in herbivores (slope = 1.036), omnivores (slope = 1.040) and piscivores (slope = 1.039), but in all three cases isometry was included within the 95% confidence intervals. Carnivores (slope = 0.983) scaled isometrically, and insectivores (slope = 0.961) scaled with negative allometry (Fig. 11B), but with isometry included within their confidence intervals (Table S23). Carnivores were only recovered as significantly different to omnivores and piscivores when the contribution of intercepts was considered, while insectivores were found to differ statistically from herbivores and omnivores, but in the latter case only when the contribution of intercepts was accounted for (Table S30). All other comparisons of trophic groups were non‐significant.

Therefore, the choice of metric (length *versus* volume) chosen to represent neck size did not impact qualitatively on the allometric patterns recovered for herbivores or piscivores, but did have a qualitative impact on that of carnivores, insectivores and omnivores (Fig. 11C).

#### (c) Head versus neck scaling

**Fig. 12:**
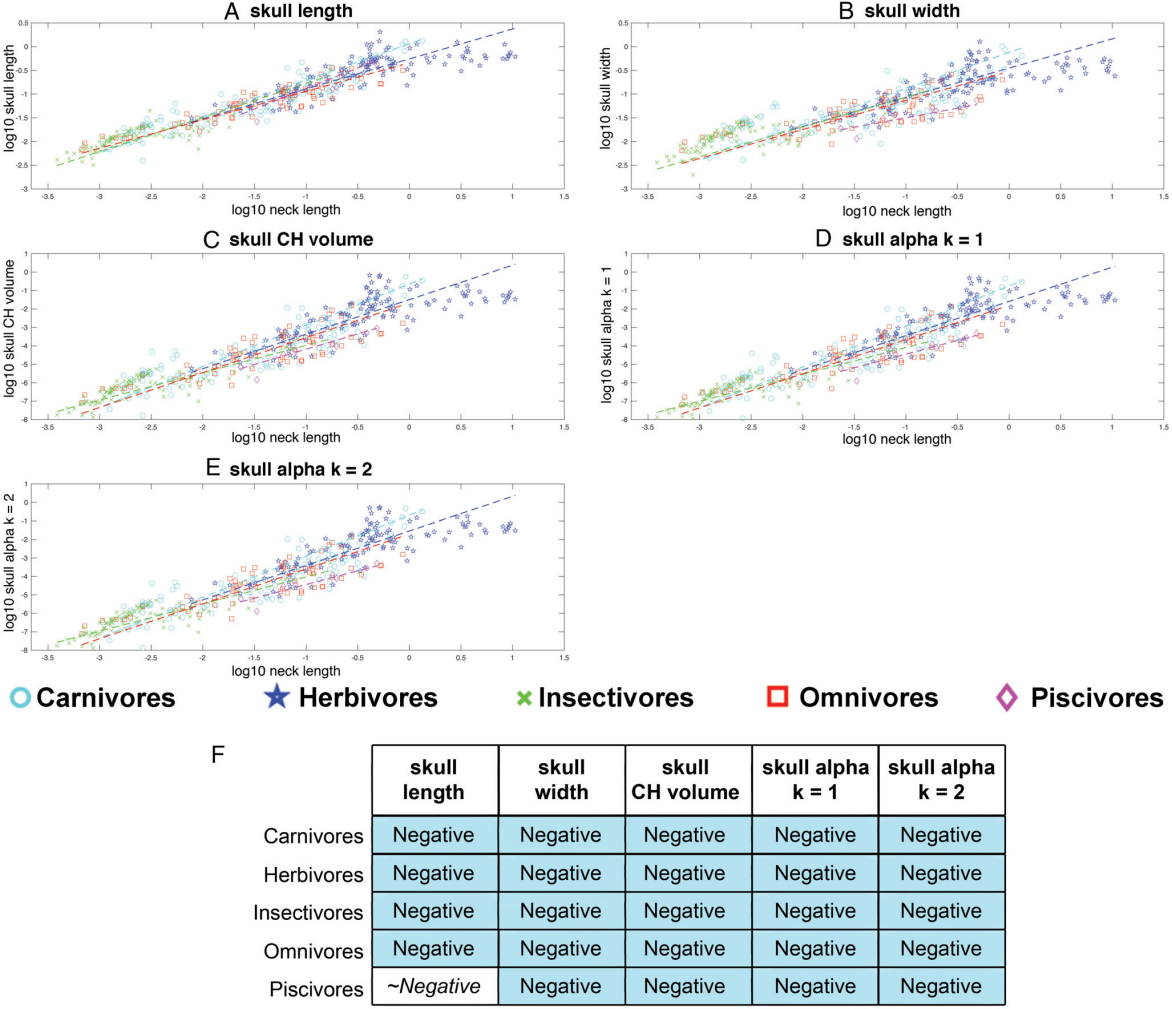
Head–neck allometry in different dietary groups. Scaling relationships between different metrics for head size and overall neck length in major tetrapod trophic groups using phylogenetically informed linear fits. The head is represented by (A) skull length, (B) skull width, (C) head convex hull (CH) volume and three α‐shape volumes where the α‐shape refinement coefficient (*k*) was (D) 1 and (E) 2. Where the head is represented by a linear metric, isometry would result in a slope of 1, while for volumetric metrics isometry would result in a slope of 3. (F) Summary of the qualitative allometric patterns for each metric, where negative allometry with isometry not included in the 95% confidence intervals is colour‐coded blue, and the white cell indicates a negative allometric slope but with isometry included within the 95% confidence intervals. The data set consisted of 124 carnivores, 120 herbivores, 94 insectivores, 64 omnivores and 8 piscivores. Full details of the regression model information can be found in Table S31. CHV, convex hull volume.

Linear relationships between neck length and all skull size metrics were statistically significant (*P* < 0.05) for all dietary categories (Fig. 12; Table S31). All metrics for all dietary categories scaled with negative allometry relative to neck length, with 95% confidence intervals below isometry for all variables except skull length in piscivores where 95% confidence intervals included isometry (Fig. 12; Table S31). Carnivores scaled closest to isometry in all skull metrics except for skull length, where piscivores showed the highest slope, which contrasts with the latter group yielding the strongest negative allometry of any dietary category for skull width relative to neck length. Omnivores and piscivores were never recovered as statistically different to each other, while the vast majority of other dietary categories were recovered as significantly different in all metrics (Tables S32–S36). Exceptions were: omnivores and insectivores not differing significantly in any metric when the contributions of intercepts were included; omnivores not differing significantly in skull width from carnivores and insectivores when the contributions of intercepts were included (Table S33); herbivores not varying statistically from piscivores and omnivores in skull width in their slopes alone, without considering intercepts (Table S33); the difference between the slopes of herbivores and carnivores, and between insectivores and those of omnivores and piscivores for skull volumes not reaching significance when intercept effects were considered, and between herbivores and piscivores when the contribution of intercepts were not considered (Tables S34–S36).

## V. DISCUSSION

It is widely accepted that a variety of intrinsic and extrinsic factors have acted upon the head and neck to shape their absolute and relative proportions across tetrapod evolution (e.g. Emerson, 1985; Emerson & Bramble, 1993; Christiansen, 1999; Goswami, 2006; Slater & Van Valkenburgh, 2009; Böhmer *et al*., 2015; Arnold *et al*., 2017; Terray *et al*., 2020; Arnold, 2021; Marek *et al*., 2021; Maher *et al*., 2022). However, linking patterns of head and neck size evolution across diverse taxonomic and trophic groupings to mechanical, physiological and ecological drivers remains difficult due to variation in the metrics used to represent head and neck size and to achieve size normalisation. Previous studies have also tended to focus on specific tetrapod subgroups. Here we analysed head and neck size across tetrapods more broadly by using several different metrics to represent the size of these body segments and a single metric for whole‐body size against which to judge absolute and relative scaling patterns. This allows a more direct comparison of relative head and neck size across major taxonomic and ecological groups, and allows us to investigate how the choice of metric used to represent head and neck size impacts on upon deductions made about allometric and ecomorphological patterns in these important body segments.

### (1) Methodological comparisons – overview

We compared head and neck size using different metrics and identified a number of instances where different metrics produced conflicting allometric patterns in head and neck size. In our analyses of allometry across our tetrapod sample as a whole (Fig. 4), the head was found to scale unequivocally with negative allometry across all five metrics for head size. However, the two neck size metrics yielded qualitatively different results, with neck volume scaling isometrically while neck length scaled with positive allometry. In our assessment of linear *versus* non‐linear scaling, we also recovered qualitatively different results across the different segment size metrics, where linear measures for head and neck size were marginally better described by linear regression models, while volumetric size metrics were slightly better described by quadratic models (Table S5). In individual taxonomic groups, the choice of metric (length *versus* volume) to represent neck size had at least some qualitative impact on the pattern recovered for all taxonomic groups (Fig. 8) and the choice of metric (length, width or volume) chosen to represent skull size impacted qualitatively on the allometric pattern recovered for other reptiles and amphibians (Fig. 7). Only in omnivores did the different head size measures unequivocally deliver the same qualitative allometric pattern across trophic groups (Fig. 10), while the metric chosen to represent neck size (length *versus* volume) also had a qualitative impact on the pattern recovered for carnivores, insectivores and omnivores (Fig. 11). In many of these cases, the relative ordering of groups in terms of the strength or magnitude of their allometric patterns also varied. The choice of metric to represent the gross size of the head and neck therefore clearly has the potential to influence macroevolutionary and ecological inferences about absolute and relative allometric patterns and subsequently the causative factors underpinning them.

The implicit cause of these differences in allometric patterns across metrics is variation in segment shape. This is manifested most simply and obviously in instances where skull length and width show qualitatively different allometric trajectories. For example, negative allometry in skull length in amphibians is coupled with positive allometry in skull width, suggesting that (on average) their skulls become shorter but wider as overall body sizes increases. Volumetric measures suggest this allometric shape change in amphibians results in either no change (isometric scaling) or a slight reduction (negative allometry) in the overall gross size of the head as overall body size increases. The opposite shape‐related pattern is qualitatively observed in piscivores, which is the only dietary category to show some evidence of positive scaling of skull length relative to overall body size (Fig. 10F, Table S23), while their skull width scales with the strongest negative allometry of any dietary category (Table S23) (although note the low sample size and broad confidence intervals for this group).

The different volumetric approaches tested here varied systematically in the extent to which they were influenced by the detailed 3D topology of the skull (Fig. 3): convex hulls utilise the least 3D topological information and are only fitted to the geometric extremes of the skull, resulting in a volume that may contain significant vacant space, while incrementally lower *k* values in our α‐shape iterations effectively increase the amount of topological (i.e. external shape) information used in the generation of the resulting volume and typically reduce the amount of vacant space (Fig. 3). Increasing the ‘tightness’ with which volumes adhered to the external topology of the skulls appeared to result in a decrease in slope (i.e. greater negative allometry) in most taxonomic and dietary categories, and in fact influenced whether amphibians could be unequivocally said to scale with negative allometry relative to overall body size (Fig. 7). This apparent reduction in slope is most likely a methodological phenomenon, resulting from the same phenomenon that led us to exclude α‐shape *k* values below 1 from our analyses (see Section III.2.*b*). This methodological issue highlights the challenges of applying automated algorithms across such a large range of body sizes, particularly where the technique used to create the original 3D meshes must also change to cope with scale differences.

The demonstration here that different measurements used in the literature to represent head and neck size yield qualitatively inconsistent results in terms of allometry and taxonomic and ecological group comparisons emphasises the caution needed when attempting to assess similarities and differences in studies that use different metrics. These findings clearly stress the need to consider which metrics are most appropriate given the research aims or questions of the study. For studies of gross body segment allometry (i.e. how overall head size scales with overall body size) we suggest that volumetric methods, in principle, fundamentally capture or represent the overall size of most body segments more holistically than a single linear measurement. Practically, this is supported by our results, with our three volumetric measurements more frequently yielding qualitatively consistent allometric results for individual taxonomic and dietary categories. However, there may be instances where linear measurements or a mixture of linear and volumetric measures are more appropriate to the questions being addressed. For example, skull (or mandible) length may be a more appropriate size metric if the goal of the study is to investigate allometric trends in the skull as a lever system (e.g. Holmes *et al*., 2024). Our results emphasise the need to consider carefully the most appropriate choice of body segment size metrics given the potential for quantitatively and qualitatively disparate allometric scaling trends.

### (2) Linear *versus* non‐linear scaling in the tetrapod head and neck

A number of allometric studies have sought to investigate whether size‐related trends in certain body segments are best described by log‐transformed linear or non‐linear changes (e.g. Economos, 1983; Silva, 1998; Campione, 2017; Maher *et al*., 2022). These analyses are often motivated by the hypothesis that non‐linear changes may indicate the existence of size thresholds in organismal form and function, about which constraints exist that dictate differential scaling in small *versus* large animals (e.g. Biewener, 1989, 2005; Christiansen, 2002). Here we find that scaling of linear measurements of gross head and neck size are marginally better described by linear regression models, while volumetric metrics are indeed slightly better described by quadratic (i.e. non‐linear) fits (Table S5). In the skull, the quadratic model fits and the results of subdividing taxa into size bins (Fig. 6) indicate that this non‐linearity relates to increasing negative allometry as overall body size increases. In the neck, however, it appears the opposite is true, with larger taxa tending towards positive allometry compared to negative or isometric scaling of neck volume in smaller taxa (Fig. 6). While our quadratic models take some account of phylogeny, it is highly likely that both these results are considerably influenced by sauropod dinosaurs. This is confirmed by the impact of excluding sauropods from OLS regressions, which resulted in slightly reduced negative allometry in skull metrics and a reduction in slopes for neck metrics (Table S2). These animals not only have the largest overall body size in our data set (and the largest body sizes ever to have evolved among terrestrial vertebrates), but also have amongst the largest neck sizes in absolute terms, and smallest head sizes relative to overall body size. Larger changes in slopes were observed when sauropods were excluded from analyses of allometry in head size relative to neck length (Table S4), reflecting the combined effects of their relatively small heads and large necks.

### (3) Taxonomic patterns in head and neck size

All our head size metrics scale with negative allometry relative to overall body size in mammals, birds and non‐avian dinosaurs (Fig. 7), providing strong evidence that the head becomes relatively smaller as body size increases in these broad taxonomic groupings. Our non‐avian dinosaur result is consistent with negative allometry recovered by Bestwick *et al*. (2022) despite the different metrics (skull length *versus* femur length) used in that study. However, our findings of negative allometry in mammals generally are difficult to compare to previous studies, which have focused on scaling in taxonomic subgroups within mammals. The negative allometry recovered for carnivorans (Van Valkenburgh 1990), ungulates and macropodoids combined (Janis 1990), and rodents (Rinderknecht & Blanco, 2008; Bertrand *et al*., 2016) in previous studies is consistent with our finding for mammals overall, despite these earlier studies using species average literature values for body mass and in some cases OLS regressions that did not account for phylogeny.

In a sample of 48 species of birds with broad phylogenetic coverage, Marek *et al*. (2021) recovered an isometric relationship between skull mass and body mass in birds, using α‐shape volume methods nearly identical to those used here to represent head size, but with a mixture of measured values and limb bone regression equations to derive specimen body masses. While this difference in overall body size metric may contribute to the disparity between our allometric findings *versus* those of Marek *et al*. (2021), we suspect that sampling may also play an important role. Marek *et al*. (2021) explicitly note considerable scatter and broad confidence intervals in their data, which is also seen here in our bird relationships (Figs 7 and 8, Table S9). Given the taxonomic, morphological and ecological diversity of extant Aves, it seems naïve to suggest that existing studies with sampling sizes of less than 50 species have provided the final word on allometric scaling of head size in birds.

As discussed above, our results suggest that width of the skull, on average, increases with body size to a greater extent than skull length in amphibians and other reptiles (Fig. 7). This correlates with other studies in other reptiles that as absolute head size increases the skull becomes relatively shorter and wider (Foth & Joyce, 2016; Gignac & O'Brien, 2016; Souza, 2021) and is comparable to previous findings in amphibians by Bardua *et al*. (2019, 2021) and Paluh *et al*. (2020). Bigger reptiles are known to have relatively wider skulls that allow relatively larger bite forces to include larger and harder prey in their diet (Herrel *et al*., 2001; Verwaijen *et al*., 2002), while wide skulls are also associated with increase gape to enable suction feeding in amphibians (Fernandez, Irish & Cundall, 2017; Bardua *et al*., 2021).

Our results suggest that as body size increases in mammals the neck becomes proportionally longer (i.e. unambiguous positive allometry) while neck volume scales only slightly above isometry with lower 95% confidence intervals that include isometry (Fig. 8). To our knowledge, only Arnold *et al*. (2017) have examined neck allometry in mammals generally and recovered slight negative scaling of neck length with body mass when regression slopes considered phylogenetic relationships (as here), but positive allometry when non‐phylogenetic regression was used. The data set of Arnold *et al*. (2017) was considerably larger than this study (352 mammalian species *versus* 143 used here) and the strong influence of phylogeny on their regression results would indirectly suggest that differential sampling could be contributing to the contrasting scaling pattern recovered here. On the other hand, Arnold *et al*. (2017) used body masses derived from an online database which were thus not matched to the specimens from which neck lengths were measured. It is therefore once again difficult to reconcile or fully explain contrasting allometric patterns recovered across different studies.

Arnold *et al*. (2017) linked their recovery of negative allometric scaling in the neck to what they interpreted in past studies to be evidence for isometric scaling of the skull recovered within various mammalian subgroups (e.g. Slater & Van Valkenburgh, 2009; Cardini & Polly, 2013; Cardini *et al*., 2015; Law *et al*., 2018; van de Geer *et al*., 2018; Cardini, 2019) to propose an intrinsic mechanical linkage between the head and neck in mammals. Specifically, they argued that relatively shorter necks compensated for relatively larger or geometrically similar heads as body size increases in mammals by minimising or reducing distance between the head's centre of mass and its centre of rotation (i.e. the cervico‐thoracic junction), thus minimising the effective first mass moment of the head. However, as noted above, the studies referenced by Arnold *et al*. (2017) and Bestwick *et al*. (2022) did not attempt to quantify head allometry with respect to body size. Where explicit assessment of head to body size allometry has been conducted in mammalian subgroups they have recovered negative allometry (Janis, 1990; Van Valkenburgh, 1990), as we do here. Interestingly, whether negative allometry in the skull relative to body size detracts from the significance of the hypothesised mechanical interaction or constraint between head and neck size depends on whether the neck length scales with slight negative allometry (Arnold *et al*., 2017) or slight positive allometry, as recovered here. If the neck scales with negative allometry relative to body size (Arnold *et al*., 2017) while relative head size also decreases then this would suggest that the mechanical linkage (and the constraint imposed by the head on neck length) is perhaps weaker than previously assumed. However, our finding of slight positive neck allometry alongside negative head allometry with respect to body size, and more directly that head size scales with negative allometry with respect to neck size, is consistent with the hypothesis that longer necks are mechanically coupled with smaller heads due to weight‐bearing demands.

Recovery of isometric scaling of neck length and head size with each other, and neck length with overall body mass in birds led Marek *et al*. (2021) to suggest this mechanical interaction or constraint inferred in mammals (Arnold *et al*., 2017) is absent in birds. Our findings are not consistent with the findings of Marek *et al*. (2021) for birds as we recover positive allometry in the avian neck relative to overall body size (Fig. 8), as well as negative allometry in skull metrics relative to neck length (Fig. 9). As noted above, relatively small sample sizes may explain these conflicting results recovered for birds. Indeed, the disparate (and seemingly adaptively significant) allometric patterns we recover across trophic groups (see Section V.4) suggests that a ‘one size fits all’ pattern for birds may not be appropriate given their ecological diversity [see Marek *et al*. (2021) for additional discussion].

Neck allometry in non‐avian dinosaurs, other reptiles and amphibians has received less attention in the literature relative to mammals and birds. Not all other reptiles linear relationships were statistically significant, which probably reflects a combination of morphological diversity and relatively small sample size used here. We found here that amphibian neck size is significantly shorter compared to all other groups at their range of body sizes, and with neck volume and length increasing in size much more slowly as body size increases than in other taxonomic groupings. This is probably due to the amphibian neck being heavily constrained to having only one cervical vertebra. Non‐avian dinosaur neck length scales isometrically overall body size (Fig. 7), which is perhaps surprising given the largest animals in this group are sauropods, which have long necks and have previously been shown to scale with strong positive allometry (Fig. 2; Bates *et al*., 2016). Isometric scaling here likely reflects the diversity of relative neck sizes observed at smaller body sizes in ornithischians and particularly carnivorous theropod dinosaurs and the tendency for PGLS to dilute the impact of sauropods (i.e. a relatively close phylogenetic grouping that dominates the larger extreme of the data set). Indeed, positive allometry (slope = 0.397) in neck length is recovered for non‐avian dinosaurs overall when non‐phylogenetic (ordinary least squares) regression is used rather than PGLS (Table S2).

### (4) Trophic patterns in head and neck size

Across our tetrapod sample as a whole, we find that skull width scales isometrically in carnivores, while skull length and volume scale with negative allometry (Fig. 10), suggesting that, on average, skulls become slightly wider and shorter in relative terms as overall body size increases in this dietary group. A relatively wider skull may facilitate higher bite force and the ability to deal with large torsional loadings from struggling prey (Radinsky, 1981, 1984; Wroe & Milne, 2007; Felice *et al*., 2021). This relationship between relative skull width/length and carnivory has been documented in amphibians (Deban, O'Reilly & Nishikawa, 2001; Vidal‐García & Keough, 2017; Bardua *et al*., 2021), non‐avian dinosaurs (King, 1996; Bates & Falkingham, 2012), other reptiles (Vanhooydonck *et al*., 2011; Vitt & Caldwell, 2014; Foth & Joyce, 2016; Gignac & O'Brien, 2016; Herrel, Petrochic & Draud, 2018; Godoy, 2020; Souza, 2021) and mammals (Radinsky, 1981, 1984; Cardini & Polly, 2013; Cardini *et al*., 2015; Marshall & Pyenson, 2019). We also recover a similar pattern in insectivorous species, which could be associated with adaptation towards capturing and crushing large insectivorous prey (Herrel *et al*., 2018).

Our results suggest that neck length increases with body size to a significantly lesser extent in carnivores than herbivores, which may to an extent reflect the disparate mechanical demands of these two feeding strategies. Shorter necks in carnivores may give the head the greater structural stability needed to capture prey (Taylor & Wedel, 2013; Felice *et al*., 2021). In herbivores, by contrast, such stability is less important, but relatively longer necks would increase the feeding envelope (Taylor & Wedel, 2013). Previous work has suggested that a shift away from carnivory may have facilitated shifts in longer neck lengths and smaller skull sizes in bird‐line dinosaurs (Zanno & Makovicky, 2011). Indeed, here we find that carnivore head size increases at a significantly greater rate in all linear and volumetric measurements than in herbivores. Smaller skull sizes in herbivores have been related to reduced dentition and/or greater food processing in the gut *versus* the mouth relative to carnivores (Sues & Reisz, 1998). The combination of relatively smaller heads and longer necks in herbivores compared to carnivores (Fig. 12) may reflect the same mechanical interaction in these body segments invoked by Arnold *et al*. (2017) to explain the possible coupling of negative allometry in neck lengths and isometric scaling of head size in mammals (see Section V.3).

Piscivores were the only trophic group found to scale with positive allometry in skull length. While 95% confidence intervals included isometry (almost certainly owing to the very small sample for this group) their slope was nevertheless statistically greater than carnivores and herbivores. Conversely, piscivore skull width scaled with the strongest negative allometry in piscivores and the group were again statistically different to carnivores and herbivores. A relatively longer skull is widely considered adaptive to increase the speeds of attack and prey capture in water associated with a diet of soft fast‐moving prey such as fish (Stayton & Ruta, 2006; Witzmann & Schoch, 2006; Maganuco & Pasini, 2009; Fortuny *et al*., 2011; Ballell *et al*., 2019). In contrast to their recovery as the most ‘extreme’ trophic group in skull length and width allometry, piscivores are unremarkable or intermediate among dietary groups in the allometric patterns recovered for the skull volume metrics. While it should be noted from the perspective of wider biological interpretations that our sample size for piscivores is small, this pattern nevertheless underlines our earlier methodological point about metric choice and the potential to miss adaptive signals through inappropriate representation of body segment size. Indeed, it suggests that in certain instances, analysis of multiple metrics may be required to capture adaptive signals fully. In the neck, both length and volume scaled with positive allometry, on average, in piscivores. Our piscivore sample is predominantly composed of fish‐eating birds and these animals have been suggested to use their neck as an important tool to manipulate the environment around them (Marek *et al*., 2021), and particularly in feeding due to the constraints of flying on the forelimb. A longer neck, in particular, would allow for reaching fish in deeper areas of water as seen in herons, spoonbills and cormorants (Wilkinson & Ruxton, 2012).

## VI. CONCLUSIONS

(1) In our sample of terrestrial tetrapods as a whole, skull volume, length and width all show negative allometry, with relative neck length increasing (positive allometry) and neck volume scaling isometrically with body size.
(2) Across our tetrapod sample as a whole, allometric patterns in linear size metrics (skull length, width and neck length) are better described by a linear model overall, whilst volumetric measurements (skull volume, neck volume and α‐shapes) fit a quadratic model better. Species over 100 kg tend to show greater negative allometry in skull volume, whilst the neck shows strong positive allometry in larger taxa.
(3) For the skull, different metrics mostly yield the same qualitative picture of allometric patterns within and across major taxonomic groups, while the choice of metric (length *versus* volume) chosen to represent neck size impacts qualitatively on the majority of allometric patterns recovered for major taxonomic groups.
(4) Only in omnivores do the different head size measures unequivocally deliver the same qualitative allometric pattern across trophic groups. The choice of metric (length *versus* volume) chosen to represent neck size does not impact qualitatively on the allometric pattern recovered for herbivores or omnivores, but does have a qualitative impact on that of carnivores, insectivores, with some ambiguity for piscivores where confidence intervals are extremely broad due to small sample size.
(5) The disparate allometric patterns given by different metrics typically result from systematic variation in segment shape, which may often have adaptive significance. For example, skull width increases to a greater extent than skull length with body size in amphibians, other reptiles and carnivores and insectivores, while the opposite pattern is seen in piscivores. These trends may represent changes in skull shape that favour bite force over velocity in larger amphibians, other reptiles and carnivores and insectivores, compared to velocity being favoured over force in piscivores.
(6) We recover negative allometry in all head size variables relative to neck length in all taxonomic and trophic groups, apart from skull length in piscivores where broad confidence intervals include positive allometry, which may be important to the use of the neck in this group use in prey capture.
(7) Neck length increases with body size to significantly lesser extent in carnivores than herbivores, while the opposite allometric pattern is seen in skull size. The combination of relatively smaller heads and longer necks in herbivores compared to carnivores may reflect the same mechanical interaction in these body segments: relatively larger heads (favouring a bigger gape and stronger bite force) in carnivores necessitates a shorter neck to minimise the first moment of mass of the head, while relatively smaller head sizes in herbivores (as food processing shifts to the gut) allows longer necks to increase the feeding envelope accessible to the head–neck system.
(8) Our results indicate that different measurements used in the literature to represent head and neck size do not always yield qualitatively consistent results in terms of allometric patterns in our tetrapod sample as a whole, or within and between major taxonomic and ecological groups. This emphasises the importance of selecting the most appropriate metric for head and neck size given the experimental goals or hypotheses under study. This underlines the potential for missing important and potentially biologically meaningful (adaptive) signals through inappropriate representation of body segment size.

## Supporting information


**Table S1.** Summary of PGLS information for log_10_‐transformed linear and volumetric parameters against body size (whole body convex hull volume) for all taxa (*N* = 410).
**Table S2.** Summary of OLS regression information for log_10_‐transformed volume parameters against body size (whole body convex hull volume) for all taxa (*N* = 410) and for the data set excluding sauropod dinosaurs (*N* = 387).
**Table S3.** Summary of PGLS information for log_10_‐transformed parameters against neck length for all taxa (*N* = 410).
**Table S4.** Summary of OLS regression information for log_10_‐transformed volume parameters against body size (whole body convex hull volume) for all taxa (*N* = 410) and for the data set excluding sauropod dinosaurs (*N* = 387).
**Table S5.** Summary of results for the linear and quadratic models for all taxa against body size (whole body convex hull volume) for all taxa (*N* = 410).
**Table S6.** Summary of PGLS information for log_10_‐transformed volume and length parameters against body size (whole body convex hull volume) for all taxa under and over 25 kg.
**Table S7.** Summary of PGLS information for log_10_‐transformed volume and length parameters against body size (whole body convex hull volume) for all taxa under and over 100 kg.
**Table S8.** Summary of PGLS information for log_10_‐transformed volume and length parameters against body size (whole body convex hull volume) for all taxa under and over 500 kg.
**Table S9.** Summary of PGLS information for log_10_‐transformed length and volume parameters against body size (whole body convex hull volume) for all taxonomic groupings.
**Table S10.** Results from a phylogenetic ANCOVA for skull length amongst different taxonomic groupings.
**Table S11.** Results from a phylogenetic ANCOVA for skull width amongst different taxonomic groupings.
**Table S12.** Results from a phylogenetic ANCOVA for skull volume amongst different taxonomic groupings.
**Table S13.** Results from a phylogenetic ANCOVA for skull α‐shape *k* = 1 amongst different taxonomic groupings.
**Table S14.** Results from a phylogenetic ANCOVA for skull α‐shape *k* = 2 amongst different taxonomic groupings.
**Table S15.** Results from a phylogenetic ANCOVA for neck length amongst different taxonomic groupings.
**Table S16.** Results from a phylogenetic ANCOVA for neck volume amongst different taxonomic groupings.
**Table S17.** Summary of PGLS information for log_10_‐transformed parameters against neck length for all taxonomic categories.
**Table S18.** Results from a phylogenetic ANCOVA for skull length *versus* neck length amongst different taxonomic groupings.
**Table S19.** Results from a phylogenetic ANCOVA for skull width *versus* neck length amongst different taxonomic groupings.
**Table S20.** Results from a phylogenetic ANCOVA for skull volume *versus* neck length amongst different taxonomic groupings.
**Table S21.** Results from a phylogenetic ANCOVA for skull α‐shape *k* = 1 *versus* neck length amongst different taxonomic groupings.
**Table S22.** Results from a phylogenetic ANCOVA for skull α‐shape *k* = 2 *versus* neck length amongst different taxonomic groupings.
**Table S23.** Summary of PGLS information for log_10_‐transformed parameters against body size (whole body convex hull volume) for all dietary categories.
**Table S24.** Results from a phylogenetic ANCOVA for skull length amongst different dietary groupings.
**Table S25.** Results from a phylogenetic ANCOVA for skull width amongst different dietary groupings.
**Table S26.** Results from a phylogenetic ANCOVA for skull volume amongst different dietary groupings.
**Table S27.** Results from a phylogenetic ANCOVA for skull α‐shape *k* = 1 amongst different dietary groupings.
**Table S28.** Results from a phylogenetic ANCOVA for skull α‐shape *k* = 2 amongst different dietary groupings.
**Table S29.** Results from a phylogenetic ANCOVA for neck length amongst different dietary groupings.
**Table S30.** Results from a phylogenetic ANCOVA for neck volume amongst different dietary groupings.
**Table S31.** Summary of PGLS information for log_10_‐transformed parameters against neck size (whole body convex hull volume) for all dietary categories.
**Table S32.** Results from a phylogenetic ANCOVA for skull length *versus* neck length amongst different dietary groupings.
**Table S33.** Results from a phylogenetic ANCOVA for skull width *versus* neck length amongst different dietary groupings.
**Table S34.** Results from a phylogenetic ANCOVA for skull volume *versus* neck length amongst different dietary groupings.
**Table S35.** Results from a phylogenetic ANCOVA for skull α‐shape *k* = 1 *versus* neck length amongst different dietary groupings.
**Table S36.** Results from a phylogenetic ANCOVA for skull α‐shape *k* = 2 *versus* neck length amongst different dietary groupings.
